# Deletion of PLCγ1 in GABAergic neurons increases seizure susceptibility in aged mice

**DOI:** 10.1038/s41598-019-54477-4

**Published:** 2019-11-28

**Authors:** Hye Yun Kim, Yong Ryoul Yang, Hongik Hwang, Ha-Eun Lee, Hyun-Jun Jang, Jeongyeon Kim, Esther Yang, Hyun Kim, Hyewhon Rhim, Pann-Ghill Suh, Jae-Ick Kim

**Affiliations:** 1 School of Life Sciences, Ulsan National Institute of Science and Technology (UNIST), Ulsan, 44919 Republic of Korea; 2 Aging Research Center, Korea Research Institute of Bioscience and Biotechnology (KRIBB), Daejeon, 34141 Republic of Korea; 3 Center for Neuroscience, Brain Science Institute, Korea Institute of Science and Technology (KIST), Seoul, 136-791 Republic of Korea; 4 Korea Brain Research Institute (KBRI), Daegu, 41062 Republic of Korea; 5 Department of Anatomy, College of Medicine, Korea University, Seoul, 136-705 Republic of Korea

**Keywords:** Epilepsy, Cellular neuroscience

## Abstract

Synaptic inhibition plays a fundamental role in the information processing of neural circuits. It sculpts excitatory signals and prevents hyperexcitability of neurons. Owing to these essential functions, dysregulated synaptic inhibition causes a plethora of neurological disorders, including epilepsy, autism, and schizophrenia. Among these disorders, epilepsy is associated with abnormal hyperexcitability of neurons caused by the deficits of GABAergic neuron or decreased GABAergic inhibition at synapses. Although many antiepileptic drugs are intended to improve GABA-mediated inhibition, the molecular mechanisms of synaptic inhibition regulated by GABAergic neurons are not fully understood. Increasing evidence indicates that phospholipase Cγ1 (PLCγ1) is involved in the generation of seizure, while the causal relationship between PLCγ1 and seizure has not been firmly established yet. Here, we show that genetic deletion of PLCγ1 in GABAergic neurons leads to handling-induced seizure in aged mice. In addition, aged *Plcg1*^*F/F*^*; Dlx5/6-Cre* mice exhibit other behavioral alterations, including hypoactivity, reduced anxiety, and fear memory deficit. Notably, inhibitory synaptic transmission as well as the number of inhibitory synapses are decreased in the subregions of hippocampus. These findings suggest that PLCγ1 may be a key determinant of maintaining both inhibitory synapses and synaptic transmission, potentially contributing to the regulation of E/I balance in the hippocampus.

## Introduction

GABAergic neurons synthesize and release the inhibitory neurotransmitter γ-aminobutyric acid (GABA) that controls the excitation of neurons in the brain. Although GABAergic inhibitory neurons constitute just 10–25% of the total number of cortical and hippocampal neurons, synaptic inhibition exerted by these neurons plays an essential role in regulating the synaptic transmission, synaptic plasticity, firing activity, and the synchronization of neurons through maintaining the appropriate excitation and inhibition balance (E/I balance)^1^. Recently, multiple lines of evidence have shown that the dysfunction of GABAergic neurons is implicated in various brain disorders, including epilepsy, autism, and schizophrenia. The alterations of population, structure, and synaptic connection in GABAergic neurons were observed in the patients and animal models of brain disorders, indicating that GABAergic inhibition is vital for the maintenance of normal functions in neural circuits^2–4^.

Epilepsy, one of the neurological disorders related to the dysfunction of GABAergic neurons, is characterized by recurrent seizures that are caused by abnormal hyperexcitation and the synchronization of neuronal activity. Approximately 60 million people worldwide suffer from epilepsy. Importantly, age-related prevalence of epilepsy in elderly people is higher than young people^5^. Unlike childhood epilepsy mostly caused by genetic mutations and developmental defects, the epilepsy in elderly people is considered to stem from the more complex interplay between genetic and environmental factors. In most cases, however, the detailed molecular and cellular mechanisms of epileptogenesis remain still unclear.

Brain-derived neurotrophic factor (BDNF) is a protein having multi-faceted roles in orchestrating development, synapse formation, and cognitive functions such as learning and memory in the brain. BDNF and its receptor TrkB (tyrosine receptor kinase B) signaling is one of the most likely candidates that are believed to contribute to the etiology of epileptogenesis. BDNF level and the phosphorylation of TrkB are upregulated in human patients as well as animal models of epileptic seizures^6–9^. Phospholipase C γ1 (PLCγ1) is a downstream enzyme activated by TrkB. Once activated, it hydrolyzes a phosphatidylinositol 4,5-bisphosphate (PIP_2_), generating diacylglycerol (DAG) and inositol 1,4,5-trisphosphate (IP_3_). These second messengers regulate diverse neuronal functions in the brain through the activation of protein kinase C (PKC) and intracellular calcium release^10,11^. In adult brain, PLCγ1 is highly expressed in cortex and hippocampus. More importantly, TrkB activation is accompanied by the elevated phosphorylation of PLCγ1 in the animal model of epilepsy^12^. Furthermore, neuron-specific TrkB conditional knockout mice show reduced seizure susceptibility in kindling epilepsy model and the disrupted interaction between TrkB and PLCγ1 prevents the generation of seizure^12–14^. Despite these findings, there remains a good deal of uncertainty about the specific role of PLCγ1 in the etiology of epileptogenesis.

Here, we investigate the cell-type specific role of PLCγ1 in the brain by targeting GABAergic neurons. GABAergic neuron-specific PLCγ1 conditional knockout mice, *Plcg1*^*F/F*^*; Dlx5/6-Cre*, exhibit the handling-induced recurrent seizures and epileptic EEG patterns when they are at least 6 months old. This occurrence of seizure is accompanied by other behavioral alterations including hypoactivity, reduced anxiety, and impaired fear memory. Although the number of GABAergic neurons is marginally affected, the expression of GABAergic synaptic markers and the number of inhibitory synaptic puncta are noticeably reduced in the hippocampal subregions of aged *Plcg1*^*F/F*^*; Dlx5/6-Cre* mice, which results in the attenuation of inhibitory synaptic transmission in CA3 area of the hippocampus. Taken together, our findings suggest that PLCγ1 may be one of principal molecular determinants modulating hippocampal circuit by maintaining the proper level of synaptic inhibition via GABAergic interneurons.

## Results

### Aged PLCγ1 conditional knockout mice exhibit the handling-induced seizures

**Figure 1:**
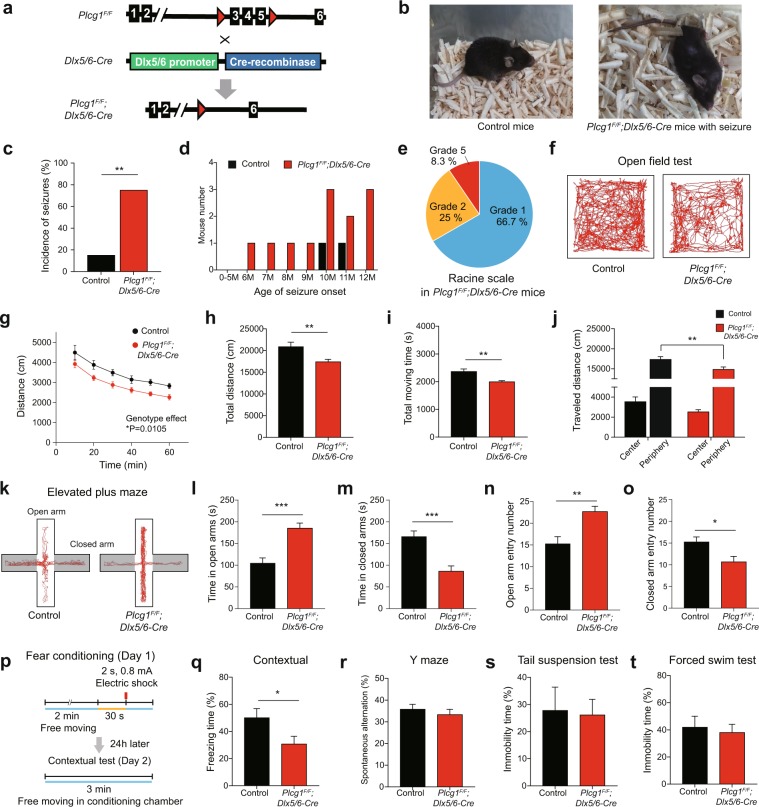
Aged *Plcg1*^*F/F*^*; Dlx5/6-Cre* mice exhibit handling-induced seizures and behavioral aberrations. **(a**) Experimental design for the generation of *Plcg1*^*F/F*^*; Dlx5/6-Cre* mice. (**b**) Normal behavior in control mice and handling-induced seizures in *Plcg1*^*F/F*^*; Dlx5/6-Cre* mice. (**c**) Percentage of seizure occurrence by the genotypes (control, 2 of 13 mice; *Plcg1*^*F/F*^*; Dlx5/6-Cre*, 12 of 16 mice; Chi-square test, **P = 0.0014). (**d**) The number of mice at age of seizure onset. (**e**) Assessment of seizure grade in *Plcg1*^*F/F*^*; Dlx5/6-Cre* mice by Racine scale. (**f**) Representative tracing of open-field test for control (n = 9) and *Plcg1*^*F/F*^*; Dlx5/6-Cre* mice (n = 11). (**g**) Distance travelled with 10 min interval in the open-field test (two-way repeated measures ANOVA, genotype effect, *P = 0.0105). (**h**) Total distance travelled in the open-field chamber (unpaired t-test, **P = 0.0077). (**i**) Time spent moving in open-field chamber (unpaired t-test, **P = 0.0012). **(j**) Thigmotaxis in the open-field test (two-way repeated measures ANOVA, genotype effect, **P = 0.0077, **P_periphery = _0.0046 in Sidak’s multiple comparison test). (**k**) Representative tracing of elevated plus maze for control (n = 12) and *Plcg1*^*F/F*^*; Dlx5/6-Cre* mice (n = 9). (**l,m**) Time spent in open (**l**) and closed arm (**m**) of elevated plus maze (unpaired t-test, ***P_open_ = 0.0002, ***P_closed_ = 0.0004). (**n**,**o**) Entry number in open (**n**) and closed arm (**o**) of elevated plus maze (unpaired t-test, **P_open_ = 0.0034, *P_closed_ = 0.0158). (**p**) Experimental scheme for contextual fear conditioning and memory. (**q**) Contextual fear memory 24 hours after conditioning (control, n = 8; *Plcg1*^*F/F*^*; Dlx5/6-Cre*, n = 11; unpaired t-test, *P = 0.0433). (**r**) Percentage of spontaneous alteration for 10 min in the Y-maze test (control, n = 8; *Plcg1*^*F/F*^*; Dlx5/6-Cre*, n = 11; unpaired t-test, P = 0.4975). (**s**) Immobility time in the tail suspension test (control, n = 8; *Plcg1*^*F/F*^*; Dlx5/6-Cre*, n = 11; unpaired t-test, P = 0.8725). (**t**) Immobility time in the forced swim test (control, n = 8; *Plcg1*^*F/F*^*; Dlx5/6-Cre*, n = 11; unpaired t-test, P = 0.7039). All data were expressed as mean ± SEM.

To determine the cell-type specific roles of PLCγ1 in the brain *in vivo*, we generated the GABAergic neuron-specific PLCγ1 conditional knockout mice. We used Dlx5/6-Cre mice in which Cre recombinase driven by Dlx5/6 promoter is expressed in most of developing and mature GABAergic neurons (Fig. 1a). Dlx5 and Dlx6 (distal-less homeobox 5 and 6 genes) are reported to be implicated in the development and migration process of GABAergic neurons^15,16^. These Dlx5/6-Cre mice were bred to *Plcg1*^*F/F*^ mice to produce *Plcg1*^*F/F*^*; Dlx5/6-Cre* mice. We confirmed the deletion of PLCγ1 in GABAergic neurons using western blotting and fluorescence *in situ* hybridization. Because the percentage of GABAergic neurons in the striatum is relatively much higher than the ones in the cortex and the hippocampus, the expression of PLCγ1 appeared significantly decreased only in the striatum compared with the cortex and the hippocampus (Fig. S1a,b). Fluorescence *in situ* hybridization data also validated the western blotting analysis, showing that the expression of PLCγ1 mRNA was reduced in the hippocampus of *Plcg1*^*F/F*^*; Dlx5/6-Cre* mice (Fig. S1c).

We first examined the behavioral consequences of cell-type specific deletion of PLCγ1 in the brain. Most notably, *Plcg1*^*F/F*^*; Dlx5/6-Cre* mice began to show recurrent behavioral seizures by routine handling stimulus when the mice reached 6 months old (Fig. 1b). 75% of aged *Plcg1*^*F/F*^*; Dlx5/6-Cre* mice exhibited seizure behaviors and the incidence of seizures was increased with age (Fig. 1c,d). Unexpectedly, handling-induced seizure was also observed from a small percentage of control mice when they were at around 10–11 months of age (Fig. 1c,d). Representative symptoms of seizure in aged *Plcg1*^*F/F*^*; Dlx5/6-Cre* mice are head nodding, and stiff body. In severe cases, losing posture, clonus of the forelimbs, and jumping were also found in *Plcg1*^*F/F*^*; Dlx5/6-Cre* mice, whereas none of these symptoms were detected in control mice (Supplementary Video 1). Using Racine scale^17^, we scored behavioral seizure grade: grade (1) stiffness and rigid posture; (2) head nodding; (3) partial forelimb clonus; (4) continuous severe whole body seizures; (5) falling, forelimb clonus and jumping (generalized motor convulsions). About 66.7% of *Plcg1*^*F/F*^*; Dlx5/6-Cre* mice exhibited mild seizures, 25% of them showed grade 2 seizures, and severe seizures were monitored in 8.3% of *Plcg1*^*F/F*^*; Dlx5/6-Cre* mice (Fig. 1e). There was, however, no significant difference in survival (or mortality) rate between control and *Plcg1*^*F/F*^*; Dlx5/6-Cre* mice (data not shown). It is important to note that seizure susceptibility induced in young adult (8–12 weeks old) mice by pilocarpine was comparable between control and *Plcg1*^*F/F*^*; Dlx5/6-Cre* mice, whereas excitatory neuron-specific PLCγ1 knockout mice (*Plcg1*^*F/F*^*; CaMKIIα-Cre* mice) displayed attenuated pilocarpine-induced seizure (Fig. S2a). These results potentially indicate that PLCγ1 may play differential roles for the generation of seizure in a cell-type-specific manner.

To interrogate other behavioral alterations induced by GABAergic neuron-specific deletion of PLCγ1, we conducted behavioral test batteries. In open field test, aged (10–16 months old) *Plcg1*^*F/F*^*; Dlx5/6-Cre* mice showed hypoactivity in locomotion and the total distance travelled was remarkably diminished (Fig. 1f–h). Moving duration in open field was also significantly reduced in *Plcg1*^*F/F*^*; Dlx5/6-Cre* mice (Fig. 1i). Interestingly, *Plcg1*^*F/F*^*; Dlx5/6-Cre* mice spent less time only in exploring the peripheral zone of the open field, possibly indicating the decreased level of anxiety (Fig. 1j). To further assess the anxiety level, we carried out the elevated plus maze (Fig. 1k). In elevated plus maze, *Plcg1*^*F/F*^*; Dlx5/6-Cre* mice spent more time in the open arms than control mice, while *Plcg1*^*F/F*^*; Dlx5/6-Cre* mice spent less time in the closed arms than control mice (Fig. 1l,m). This altered anxiety was also replicated in the entry number to each arm (Fig. 1n,o). These behavioral phenotypes are clearly indicative of low anxiety in *Plcg1*^*F/F*^*; Dlx5/6-Cre* mice. We also checked locomotive and anxiety behaviors in young adult (8–12 weeks old) mice. In young age, locomotion was slightly attenuated in *Plcg1*^*F/F*^*; Dlx5/6-Cre* mice and total moving time in open field was only statistically different between the genotypes (Fig. S2b–d). Meanwhile, the reduction of exploration in the peripheral zone of open field was detected again in young *Plcg1*^*F/F*^*; Dlx5/6-Cre* mice (Fig. S2e). Consistent with this result, young *Plcg1*^*F/F*^*; Dlx5/6-Cre* mice began to exhibit decreased anxiety in elevated plus maze (Fig. S2f–i).

Previous studies have implicated PLCγ1 with the regulation of epileptogenesis in the hippocampus. To examine the possible role of PLCγ1 in the hippocampus, we performed contextual fear conditioning, which is one form of the hippocampus-dependent learning. Contextual fear memory was markedly impaired 24 hours after conditioning in aged *Plcg1*^*F/F*^*; Dlx5/6-Cre* mice (Fig. 1p,q), while there was no difference between the genotypes in working memory that was independent of hippocampal functions (Fig. 1r). Behavioral immobility, which is considered to reflect either behavioral despair or stress-coping strategy, was not affected by GABAergic neuron-specific deletion of PLCγ1 (Fig. 1s,t). Taken together, these data point to a possible role of PLCγ1 in modulating GABAergic neurons and its malfunction may cause seizures and other behavioral alterations that are either hippocampus-dependent or hippocampus-independent.

### Epileptic EEG patterns are detected in aged *Plcg1*^*F/F*^*; Dlx5/6-Cre* mice

**Figure 2:**
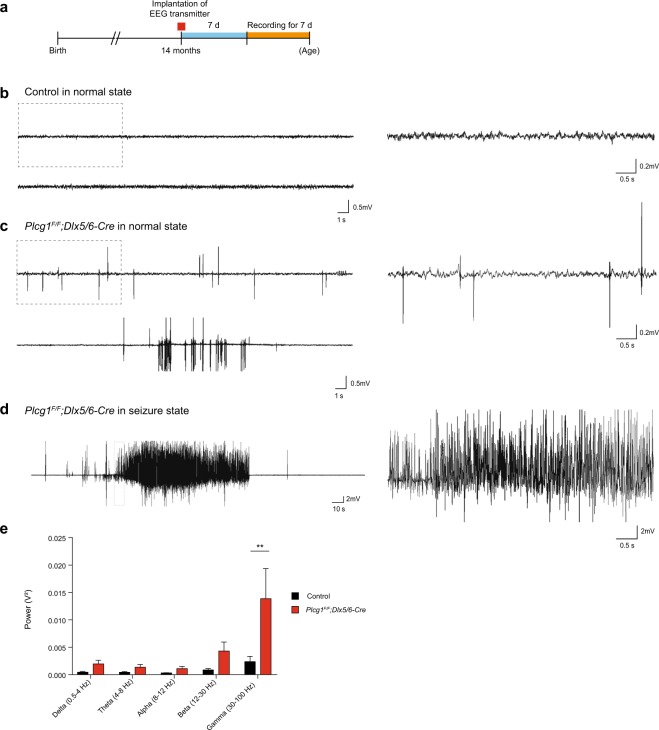
Aged *Plcg1*^*F/F*^*; Dlx5/6-Cre* mice show epileptic EEG patterns. (**a**) Schematic diagram of the EEG experimental procedure. (**b**) Representative recording traces of normal EEG patterns in control mice (left, 30 s) and temporal expansion of the same recording trace in dotted square (right, 6 s). (**c**) Representative traces of abnormal EEG patterns in freely moving *Plcg1*^*F/F*^*; Dlx5/6-Cre* mice without handling stimulus (left, 30 s) and temporal expansion of the same recording trace in dotted square (right, 6 s). (**d**) Typical epileptic EEG activity in *Plcg1*^*F/F*^*; Dlx5/6-Cre* mice (left, 5 min) and temporal expansion of the same recording trace in dotted square (right, 6 s). (**e**) EEG band power during awake state for 1 hour in non-behavioral seizure epochs without handling stimulus (control, n = 4; *Plcg1*^*F/F*^*; Dlx5/6-Cre*, n = 6; two-way repeated measures ANOVA, genotype effect, *P = 0.0169, **P = 0.0054 in Sidak’s multiple comparison test). All data were expressed as mean ± SEM.

Electroencephalogram (EEG) is usually used to detect the characteristic patterns of epileptic seizures in both human patients and animal models. We next sought to determine whether GABAergic neuron-specific deletion of PLCγ1 contributes to the generation of epileptic EEG signals. To monitor EEG waveforms, we positioned EEG electrodes directly on the dura mater overlying the hippocampus from each hemisphere. After the recovery from electrode implantation, EEG recording was conducted for 7 days in freely moving mice (Fig. 2a). During the recording period, control mice displayed normal EEG signal, lacking any signs of epileptic EEG patterns (Fig. 2b). In aged *Plcg1*^*F/F*^*; Dlx5/6-Cre* mice, however, abnormal EEG patterns including high frequency waveforms were detected even when there was no behavioral seizure. Of note, these aberrant waveforms were also observed without handling stimulus, suggesting that physical stimulus is not a prerequisite for the generation of epileptic EEG abnormalities in *Plcg1*^*F/F*^*; Dlx5/6-Cre* mice (Fig. 2c). With the manifestation of seizures, we could also observe typical seizure EEG in *Plcg1*^*F/F*^*; Dlx5/6-Cre* mice (Fig. 2d). Next, we conducted the power spectrum analysis of EEG waveforms to characterize the types of seizure. EEG signals were divided into several frequency bands via fast Fourier transformation (FFT): delta (0.5–4 Hz), theta (4–8 Hz), alpha (8–12 Hz), beta (12–30 Hz), gamma (30–100 Hz) frequency. These frequency bands have been known to be altered in various pathological conditions. Previous studies reported that high frequency EEG signal such as gamma oscillations are detected from both epilepsy patients and animal models^18,19^. Moreover, it was shown that gamma oscillation is regulated by GABAergic interneurons whose dysfunction could result in abnormal gamma oscillation patterns^20^. Akin to these findings, the analysis of EEG power spectrum revealed that the power of gamma frequency band was highly increased in aged *Plcg1*^*F/F*^*; Dlx5/6-Cre* mice, while there was no difference between the genotypes in other frequency bands (Fig. 2e). Currently, it is still controversial whether the enhanced high-frequency wave in gamma range can directly cause epileptic seizures or is just one of various symptoms in epilepsy. This result indicates that the dysfunction of GABAergic neurons by lack of PLCγ1 could induce the epileptic EEG phenotypes in aged *Plcg1*^*F/F*^*; Dlx5/6-Cre* mice, likely through increased gamma oscillation.

### The number of GABAergic synapses is markedly decreased in the hippocampus of aged *Plcg1*^*F/F*^*; Dlx5/6-Cre* mice

**Figure 3:**
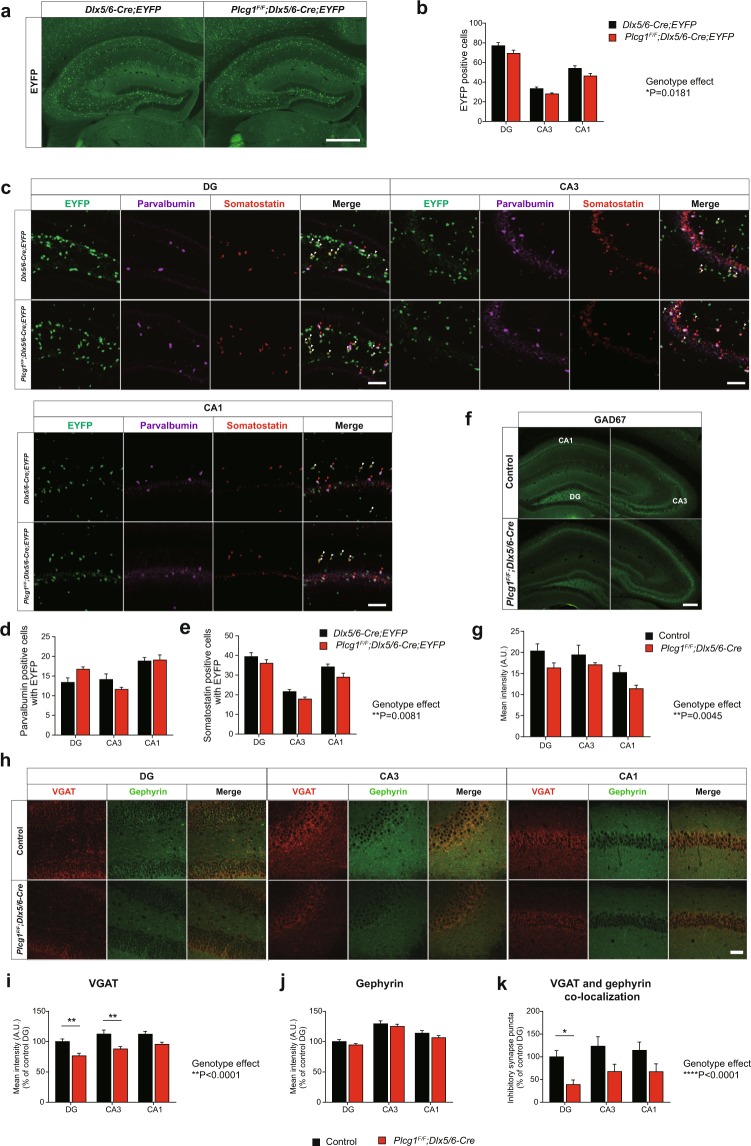
Expression of GABAergic molecules and inhibitory synaptic puncta are markedly decreased in aged *Plcg1*^*F/F*^*; Dlx5/6-Cre* mice. (**a**) Representative confocal images of EYFP positive cells in the hippocampus. Scale bar: 500 μm. (**b**) Quantification of the number of GABAergic neurons in the hippocampus (control, n = 34 slices from 6 mice; *Plcg1*^*F/F*^*; Dlx5/6-Cre*, n = 36 slices from 6 mice; two-way repeated measures ANOVA, genotype effect, *P = 0.0181). (**c**) Representative confocal images of PV and SST expressing neurons co-localized with EYFP in hippocampal subregions. Red arrowhead: co-localization of PV and EYFP. White arrowhead: co-localization of SST and EYFP. Scale bar: 100 μm. (**d**) Co-localization of PV and EYFP positive cells (n = 18 slices from 3 mice per genotype; two-way repeated measures ANOVA, genotype effect, P > 0.05). (**e**) Co-localization of SST and EYFP positive cells in the hippocampus (n = 18 slices from 3 mice per genotype; two-way repeated measures ANOVA, genotype effect, **P = 0.0081). **(f)** Representative confocal images of GAD67 expression in the hippocampus. Scale bar: 200 μm. (**g**) Quantification of the intensity of GAD67 in the hippocampus (control, n = 5 slices from 4 mice; *Plcg1*^*F/F*^*; Dlx5/6-Cre*, n = 6 slices from 3 mice; two-way repeated measures ANOVA, genotype effect, **P = 0.0045). (**h**) Representative confocal images of VGAT and gephyrin immunoreactivity in each sub-region of hippocampus. Scale bar: 50 μm. (**i, j**) Quantification of VGAT and gephyrin immunoreactivity (control, n = 28 slices from 6 mice; *Plcg1*^*F/F*^*; Dlx5/6-Cre*, n = 20 slices from 6 mice; VGAT, two-way ANOVA, genotype effect, ****P_VGAT_ < 0.0001, **P_DG_ = 0.0037, **P_CA3_ = 0.0027 by post-hoc test; gephyrin, two-way ANOVA, genotype effect, P > 0.05). (**k**) Normalized inhibitory synaptic puncta quantified by the co-localization of VGAT and gephyrin (control, n = 28 slices from 6 mice; *Plcg1*^*F/F*^*; Dlx5/6-Cre*, n = 24 slices from 6 mice; two-way ANOVA, genotype effect, ****P < 0.0001, *P = 0.0260 in DG by post-hoc test). All data were expressed as mean ± SEM.

Altered expression of key molecules or loss of cell number in hippocampal GABAergic interneurons were observed in the animal models of epilepsy and human hippocampal epilepsy^21,22^. To search for the underlying circuit mechanisms behind the seizure and epileptic EEG patterns in aged *Plcg1*^*F/F*^*; Dlx5/6-Cre* mice, we investigated the possible changes of GABAergic interneurons in the hippocampus. First, we examined any gross structural abnormalities in the hippocampus of *Plcg1*^*F/F*^*; Dlx5/6-Cre* mice by NeuN immunostaining. There was no overall changes in the hippocampus between the genotypes (Fig. S3). Owing to the lack of available PLCγ1 antibody for immunohistochemistry, we used EYFP reporter mice (R26R-EYFP) by crossing with *Plcg1*^*F/F*^*; Dlx5/6-Cre* mice to track GABAergic neurons in which PLCγ1 was knock-outed. The loss of GABAergic interneurons could be measured by counting the number of EYFP-positive neurons in each sub-region of hippocampus such as dentate gyrus (DG), CA3, and CA1. According to publicly-available single cell RNA sequencing database (http://interneuron.mccarrolllab.org), *Plcg1* is mainly expressed in parvalbumin (PV)- and somatostatin (SST)-positive interneurons, while vasoactive intestinal peptide (VIP) and other GABAergic interneuronal subtypes exhibit relatively weak expression in the mouse brain. In the absence of PLCγ1, the number of GABAergic interneurons were marginally affected in the sub-regions of hippocampus (Fig. 3a,b). We next checked whether the loss of PLCγ1 had a differential effect on the numbers of a specific subtype of GABAergic neurons in the hippocampus. Double-immunostaining with antibodies to PV and SST revealed that the number of PV- and EYFP-double positive neurons was comparable between control and *Plcg1*^*F/F*^*; Dlx5/6-Cre;EYFP* mice across the hippocampal sub-regions (Fig. 3c,d). However, SST- and EYFP-double positive neurons were slightly decreased in each sub-region of hippocampus of *Plcg1*^*F/F*^*; Dlx5/6-Cre;EYFP* mice, although statistical significance was not reached in post-hoc test (Fig. 3c,e). We also examined the altered expression of GAD67, which is a major enzyme catalyzing the synthesis of GABA in most of inhibitory neurons. In parallel with the number of GABAergic neurons, the expression of GAD67 was only slightly diminished throughout the sub-regions of hippocampus in aged *Plcg1*^*F/F*^*; Dlx5/6-Cre* mice (Fig. 3f,g).

At the local circuit level, GABAergic neuron exerts its diverse functions via inhibitory synapses. To assess any changes in the synaptic proteins of GABAergic neurons, we stained both VGAT (vesicular GABA transporter) as a presynaptic marker and gephyrin as a postsynaptic marker for inhibitory synapses. The expression of VGAT was remarkably reduced, especially in DG and CA3 of the hippocampus in aged *Plcg1*^*F/F*^*; Dlx5/6-Cre* mice, whereas the immunoreactivity of gephyrin was similar between the genotypes across the hippocampal subregions (Fig. 3h–j). We further quantified the number of inhibitory synapses by counting the synaptic puncta co-localized with VGAT and gephyrin. GABAergic synaptic puncta were also considerably decreased in the hippocampus of *Plcg1*^*F/F*^*; Dlx5/6-Cre* mice, mainly in DG (P < 0.05 in post-hoc test) and CA3 region (P = 0.0538 in post-hoc test) (Fig. 3k). These results clearly indicate that the genetic deletion of PLCγ1 only marginally affect the number of GABAergic interneurons and possibly GABA synthesis in the hippocampus, while inhibitory synaptic puncta as well as presynaptic proteins in GABAergic synapses are markedly diminished in aged *Plcg1*^*F/F*^*; Dlx5/6-Cre* mice.

### Hippocampal inhibitory synaptic transmission is attenuated in the CA3 of aged *Plcg1*^*F/F*^*; Dlx5/6-Cre* mice

**Figure 4:**
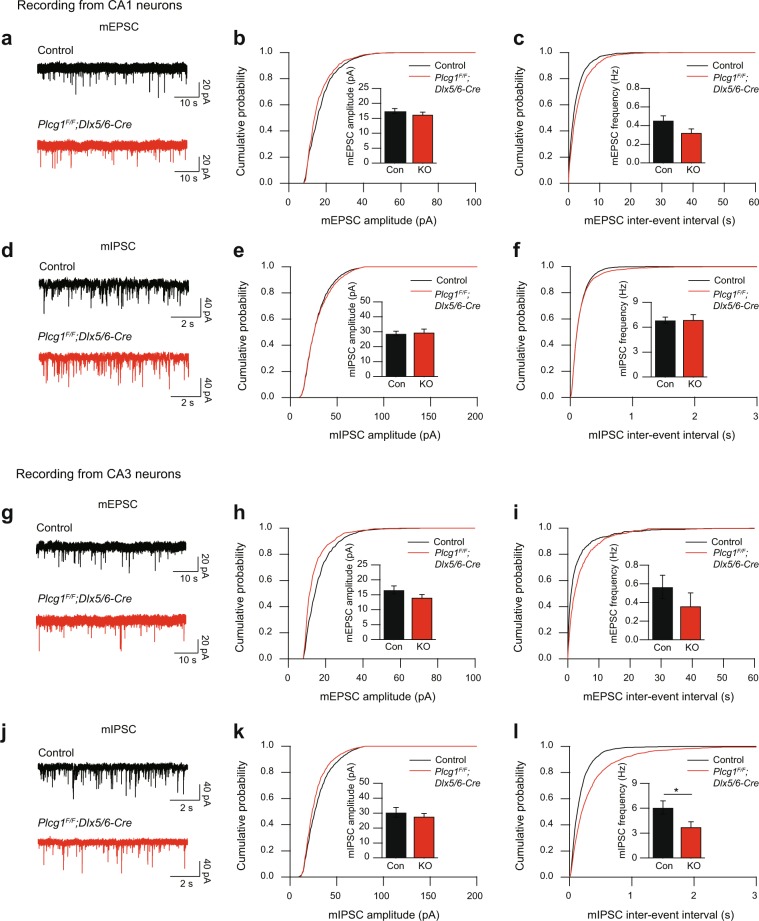
Inhibitory synaptic transmission in hippocampal CA3 region is reduced in aged *Plcg1*^*F/F*^*; Dlx5/6-Cre* mice. (**a**) Representative recording trace of mEPSCs recorded from hippocampal CA1 region in control and *Plcg1*^*F/F*^*; Dlx5/6-Cre* mice (control, n = 12, *Plcg1*^*F/F*^*; Dlx5/6-Cre*, n = 9). (**b,c**) Quantification of mEPSCs amplitude (**b**) and frequency (**c**) in CA1. (**d**) Representative recording trace of mIPSCs recorded from hippocampal CA1 region in control and *Plcg1*^*F/F*^*; Dlx5/6-Cre* mice (control, n = 10, *Plcg1*^*F/F*^*; Dlx5/6-Cre*, n = 12). (**e**,**f**) Quantification of mIPSCs amplitude (**e**) and frequency (**f**) in CA1. (**g**) Representative recording trace of mEPSCs recorded from hippocampal CA3 region in control and *Plcg1*^*F/F*^*; Dlx5/6-Cre* mice (control, n = 8, *Plcg1*^*F/F*^*; Dlx5/6-Cre*, n = 6). (**h, i**) Quantification of mEPSCs amplitude (**h**) and frequency (**i**) in CA3. (**j**) Representative recording trace of mIPSCs recorded from hippocampal CA3 region in control and *Plcg1*^*F/F*^*; Dlx5/6-Cre* mice (control, n = 6, *Plcg1*^*F/F*^*; Dlx5/6-Cre*, n = 9). (**k,l**) Quantification of mIPSCs amplitude (**k**) and frequency (**l**) in CA3 (frequency, unpaired t-test, *P = 0.0379). All data were expressed as mean ± SEM.

The reductions of inhibitory presynaptic protein and synaptic puncta may point to a functional compromise at hippocampal synapses in aged *Plcg1*^*F/F*^*; Dlx5/6-Cre* mice. It is important to note, however, that molecular and structural changes at synapses do not necessarily involve functional changes. With the above immunohistochemical data in mind, we then sought to find a functional consequence of the changes in the number of inhibitory synapses in the hippocampus of aged *Plcg1*^*F/F*^*; Dlx5/6-Cre* mice. To pinpoint the functional aberrations in the hippocampus, we conducted the whole cell patch-clamp recording and recorded the spontaneous miniature excitatory postsynaptic currents (mEPSCs) and the spontaneous miniature inhibitory postsynaptic currents (mIPSCs) from pyramidal neurons in CA1 and CA3 regions. We found that spontaneous excitatory synaptic transmission were comparable between control and *Plcg1*^*F/F*^*; Dlx5/6-Cre* mice in CA1 pyramidal neurons, exhibiting no difference in the amplitude and frequency of mEPSCs (Fig. 4a–c). In addition, there was no difference in the amplitude and frequency of mIPSCs in CA1 region, either (Fig. 4d–f). Importantly, however, the frequency of mIPSCs in CA3 region was significantly decreased in aged *Plcg1*^*F/F*^*; Dlx5/6-Cre* mice compared to control mice without any change in the amplitude (Fig. 4j–l). On the other hand, both the amplitude and frequency of mEPSCs were normal in CA3 pyramidal neurons of *Plcg1*^*F/F*^*; Dlx5/6-Cre* mice, suggesting that functional change in synaptic transmission is specific to inhibitory synapses in CA3 region (Fig. 4g–i). The reduction of mIPSCs frequency in CA3 region indicates either a decrease in release probability or inhibitory synapse loss. We next counted the number of GABAergic synaptic puncta at each layer from CA3 region to gauge layer-specific alterations of inhibitory synaptic puncta in *Plcg1*^*F/F*^*; Dlx5/6-Cre* mice. We discovered that GABAergic synaptic puncta were primarily reduced in stratum pyramidale (s.p.) and stratum oriens (s.o.) in CA3 of aged *Plcg1*^*F/F*^*; Dlx5/6-Cre* mice, whereas the number of GABAergic synaptic puncta in stratum lucidum (s.l.) and stratum radiatum (s.r.) was comparable between control and *Plcg1*^*F/F*^*; Dlx5/6-Cre* mice (Fig. S4a,b). These results demonstrate that both somatic inhibitory synapses at stratum pyramidale and dendritic inhibitory synapses at stratum oriens where basal dendrites of CA3 pyramidal neurons are located are selectively reduced in *Plcg1*^*F/F*^*; Dlx5/6-Cre* mice.

To verify whether this attenuation of synaptic inhibition in *Plcg1*^*F/F*^*; Dlx5/6-Cre* mice is age-dependent, we performed the same whole cell patch-clamp recording from hippocampal CA1 and CA3 regions in young adult (8 weeks old) *Plcg1*^*F/F*^*; Dlx5/6-Cre* mice. Most of measurements from mEPSC and mIPSC recordings were similar between the genotypes (Fig. S5). Interestingly, however, young *Plcg1*^*F/F*^*; Dlx5/6-Cre* mice showed increased amplitude of mIPSC only in CA1 region compared with control mice, indicating that genetic deletion of PLCγ1 may cause distinct effects on hippocampal GABAergic neurons with age-dependent manner (Fig. S5d,e). Considering our immunohistochemical findings described above, these results demonstrate that the defect of PLCγ1 signaling in GABAergic interneurons could attenuate inhibitory synaptic transmission via selective GABAergic synapse loss in the CA3 region of hippocampus in aged *Plcg1*^*F/F*^*; Dlx5/6-Cre* mice.

## Discussion

PLCγ1 is a crucial signal transducer and involved in various neuronal functions. However, the detailed molecular and cellular functions of PLCγ1 *in vivo* has not been fully understood in the brain. Several studies have indirectly proposed the plausible role of PLCγ1 in GABAergic neuron. TrkB signaling, the upstream pathway of PLCγ1, is reported to regulate the survival of newborn GABAergic neurons in the striatum^23^. In addition, the gene expression of GAD 65, a GABA-synthesizing enzyme localized at axon terminals, involves CREB-dependent BDNF/TrkB signaling in cortical neurons^24^. As GABAergic neurons play essential roles in shaping excitatory synaptic information and regulating neuronal activity across the brain, it is very likely that PLCγ1 in GABAergic neurons may be also implicated in the regulation of GABAergic inhibition in the brain. In this study, we aim to determine the cell-type specific roles of PLCγ1 on neural functions, especially focusing on GABAergic neurons. To achieve this goal and identify the function of PLCγ1 *in vivo*, we generated the GABAergic neuron-specific PLCγ1 knockout mice by crossing Dlx5/6-Cre mice with the mice harboring the floxed-PLCγ1 allele. Most notably, routine handling stimulus to these mice led to the recurrent seizures in an age-dependent manner, which were accompanied by epileptic EEG signals and elevated gamma frequency band. Previously, we have studied other PLCγ1 conditional knockout mice including *Plcg1*^*F/F*^*; Nestin-Cre* (Neuronal cell-specific) and *Plcg1*^*F/F*^*; CaMKIIα-Cre* (excitatory neuron-specific). None of these mice exhibited any signs of handling-induced seizures, demonstrating that selective loss of PLCγ1 in GABAergic neurons is specifically related to the generation of seizures. Interestingly, the small number of aged control mice also showed mild signs of seizure at 10–11 months of age. It is unclear what may account for this observation, but this mild seizure from old control mice might be caused by aging as aging itself is associated with increased susceptibility for some types of seizure in rodents including C57BL6 mice^25–27^. Furthermore, frequent and continuous handling we used to induce seizure at daytime might be stressful to mice, leading to stress-induced seizure in some of aged control mice^28,29^.

In addition to handling-induced seizures, other anomalous behaviors were observed in aged *Plcg1*^*F/F*^*; Dlx5/6-Cre* mice. They displayed hypo-locomotion, reduced anxiety, and the deficit in contextual fear memory. Among these changes, reduced anxiety and attenuated freezing behavior differ from the typical phenotypes of epileptic mice mostly exhibiting heightened anxiety and increased freezing, implicating that aged *Plcg1*^*F/F*^*; Dlx5/6-Cre* mice are different from common epileptic mouse models^30,31^. In general, anxiety and contextual fear memory are linked to the functions of hippocampus and GABAergic dysfunction in the hippocampus could result in comparable behavioral phenotypes from other mouse models^32–35^. However, these concomitant behavioral alterations in *Plcg1*^*F/F*^*; Dlx5/6-Cre* mice may be caused by structural and functional changes from multiple brain regions because genetic deletion of PLCγ1 is not limited to the hippocampus. In a similar vein, the hypo-locomotion of *Plcg1*^*F/F*^*; Dlx5/6-Cre* mice could be explained by GABAergic dysfunction in the striatum, of which the principal neurons are GABAergic and mediate locomotive behaviors. Thus, the defects in the hippocampus and other brain regions rendered by GABAergic neuron-specific loss of PLCγ1 may account for the alterations in anxiety level and contextual fear memory.

At the cellular level, the total number of hippocampal GABAergic interneurons and expression of GAD67 were only marginally decreased in aged *Plcg1*^*F/F*^*; Dlx5/6-Cre* mice. In addition, SST-positive interneurons were slightly diminished across DG-CA3-CA1 regions, while there was no change in the number of PV-positive interneurons in the hippocampus of *Plcg1*^*F/F*^*; Dlx5/6-Cre* mice. Given that sub-regional patterns of reductions between total GABAergic neurons and SST-positive neurons in *Plcg1*^*F/F*^*; Dlx5/6-Cre* mice are analogous in the hippocampus, marginal decline in hippocampal GABAergic neuron by the deletion of *Plcg1* could stem mostly from the loss of SST-positive neurons. Meanwhile, the expression of inhibitory presynaptic molecule, VGAT, was significantly attenuated in *Plcg1*^*F/F*^*; Dlx5/6-Cre* mice, primarily in DG and CA3 regions of hippocampus. Interestingly, the expression pattern of an inhibitory postsynaptic molecule, gephyrin, was similar between control and *Plcg1*^*F/F*^*; Dlx5/6-Cre* mice. Most importantly, the number of GABAergic synaptic puncta was considerably reduced in the hippocampus by the cell type-specific deletion of PLCγ1. Although not studied well in humans, low expression level of VGAT is associated with experimental animal models of epilepsy^36–38^. As hippocampus plays a central role in the generation of epileptic seizures, the loss of PLCγ1, especially in hippocampal GABAergic neurons, might cause the attenuation of GABAergic inhibition at hippocampal synapses.

Strengthening this idea, aged *Plcg1*^*F/F*^*; Dlx5/6-Cre* mice exhibited epileptic EEG abnormalities *in vivo* including high frequency waveforms. At synaptic level, our electrophysiological results from acute brain slices confirmed that inhibitory synaptic transmission was markedly compromised especially at CA3 of the hippocampus. Analysis of high-magnification images from distinct hippocampal layers at CA3 further demonstrated that GABAergic synaptic puncta was especially decreased in stratum pyramidale and stratum oriens in aged *Plcg1*^*F/F*^*; Dlx5/6-Cre* mice. From these findings, it seems reasonable to think that impaired synaptic inhibition measured by mIPSC at CA3 in *Plcg1*^*F/F*^*; Dlx5/6-Cre* mice could be due to the reduction of GABAergic synapses at both somatic and dendritic sites. It is important to note that, unlike aged mice, the amplitude of mIPSC at CA1 was increased by the genetic deletion of *Plcg1* in young adult *Plcg1*^*F/F*^*; Dlx5/6-Cre* mice. This finding suggests that PLCγ1 might have temporally distinct roles in regulating GABA-mediated synaptic inhibition in the hippocampus with age-dependent manner. In young animal, irrespective of the possible role of PLCγ1 in epileptogenesis, PLCγ1 might be involved in regulating the expression of inhibitory postsynaptic molecules such as gephyrin and GABA_A_ receptor. Together, our results highlight an essential role of PLCγ1 in maintaining normal GABAergic neuronal functions in the hippocampus. Moreover, the dysfunction of PLCγ1 may cause deficits in inhibitory synaptic transmission and raise the susceptibility of seizure in aged mice, potentially leading to behavioral seizure by handling-stimulus.

Some unanswered questions remain in our study. First, it is intriguing to speculate why the handling-induced seizures in *Plcg1*^*F/F*^*; Dlx5/6-Cre* mice show age-related onset. Behavioral seizure in *Plcg1*^*F/F*^*; Dlx5/6-Cre* mice occurred over 6 months of age up to 14 months old. This age range is approximately comparable to 38–47 years old of middle-aged human. Late-onset seizures can be caused by complex factors such as brain damage, infection, aging, and environment, whereas specific molecular mechanisms underlying this seizure generation is mostly unclear^39,40^. Recently, a couple of papers have described the late-onset seizures in several genetic KO mice^41,42^, yet linkage between PLCγ1 and these genes remains unexplored. Previous studies suggest that GABA-mediated inhibition can become compromised with ageing. Loss of GABAergic neurons, weakened synthesis of GABA, and impaired inhibitory synaptic transmission were all observed in aged rodents and humans^43–48^. Besides, seizure susceptibility to hippocampal CA3 kindling was elevated in aged mice^26^. Although we have no satisfactory explanation for adult onset seizure, it is possible that malfunction of GABAergic neurons caused by the lack of PLCγ1 could not induce the critical defects in the developmental and early postnatal stages, but as *Plcg1*^*F/F*^*; Dlx5/6-Cre* mice get older, the damages given to GABAergic neurons would be accelerated and gradually accumulated in hippocampal circuit. When these accumulated damages reach a certain threshold, it might result in the attenuation of synaptic inhibition through reduced GABAergic tone in the hippocampus in middle age and more elderly brain^49^.

Second, the detailed molecular mechanisms causally linking PLCγ1 to GABAergic neurons and epileptogenesis are not yet clear. Previously, several studies have focused on the role of PLCγ1 for epileptogenesis in excitatory neurons. In temporal lobe epilepsy model, hyperexcitation of excitatory neurons were induced by chemoconvulsants such as pilocarpine and kainic acid that activate muscarinic acetylcholine receptors and the glutamatergic receptors respectively^50^. This abnormal hyperexcitation of neurons caused the activation of cellular signaling pathways, including the elevated phosphorylation of PLCγ1^12,14^. Indeed, we found that excitatory neuron-specific deletion of PLCγ1 lowered the susceptibility for pilocarpine-induced seizure in *Plcg1*^*F/F*^*; CaMKIIα-Cre* mice. Converging lines of evidence suggest that BDNF/TrkB signaling is a major candidate pathway of epileptogenesis, which is the upstream of PLCγ1. Most of these studies focused on the cellular functions of BDNF/TrkB signaling in excitatory neurons. For instance, BDNF level and activation of TrkB in hippocampal excitatory neurons were increased in animal models of epilepsy and human patients^6,8,51,52^. However, BDNF signaling can also affect GABA-mediated inhibition via inhibitory synapses. It has been documented that the treatment of BDNF facilitates gephyrin clustering that stabilizes glycine and GABA_A_ receptors at inhibitory synapse^53^. In line with this report, BDNF also augments the transcription of GABA_A_ receptor subunit and expression of gephyrin^54^. Furthermore, PLC-dependent BDNF/TrkB pathway can exert temporally differential modulation on GABA_A_ receptor-mediated transmission in excitatory neurons^55^. As with our electrophysiological data, it is plausible that BDNF/TrkB signaling, potentially via PLCγ1, can have distinct and opposite roles in regulating GABA-mediated inhibition and epileptogenesis with age-dependent manner. Yet the possible involvement of BDNF/TrkB signaling in coordinating GABAergic neuronal functions and epileptogenesis is still elusive^56,57^.

Another candidate pathway mediating PLCγ1 signaling is a neuregulin 1 (NRG1) and its receptor ErbB4-related signaling pathway. Neuregulin 1, first identified as a schizophrenia risk gene, is a trophic factor containing EGF (epidermal growth factor)-like domain. This protein has been shown to function primarily in neural development and synapse formation. Of particular note is the fact that ErbB family is a tyrosine receptor kinase that can also recruit PLCγ1-related signaling. Dysfunction of this NRG1/ErbB4 signaling in GABAergic neurons has been reported to induce epilepsy and psychiatric disorders in human where single nucleotide polymorphisms of these proteins were closely linked to E/I imbalance^58–60^. Although the exact molecular mechanisms behind the defective GABAergic functions remain open questions, it is very likely that PLCγ1 might function as a convergence point from various receptor tyrosine kinases and the specific signaling pathway working on GABAergic neurons could regulate the appropriate GABAergic inhibition via the activation of PLCγ1.

In the current work, we uncovered the physiological role of PLCγ1 in GABAergic neuron *in vivo* by utilizing *Plcg1*^*F/F*^*; Dlx5/6-Cre* mice. Our findings suggest that PLCγ1 may be one of principal molecular players in regulating GABA-mediated synaptic inhibition, especially in hippocampal GABAergic neurons, whose dysfunction may result in epileptogenesis in aged mice. As the research about age-related neurological disorders becomes more and more important than ever before, our study will offer a new perspective on the cell-type specific molecular mechanisms of epileptogenesis in middle-to-old age. Undoubtedly, future studies will further examine the detailed molecular mechanism via which PLCγ1 exerts its effects on GABAergic neurons.

## Methods

### Animals

*Plcg1*^*F/F*^ mice (C57BL/6J background) were generated as previously described^61^. To generate GABAergic neuron-specific PLCγ1 knockout mice, *Dlx5/6-Cre* mice (Jax mouse cat No.008199) were crossed with *Plcg1*^*F/F*^ mice to produce *Plcg1*^*F/F*^*; Dlx5/6-Cre* mice. *Plcg1*^*F/F*^ littermate mice were used as controls in most of experiments except EYFP immunohistochemistry (Fig. 3a–e). To label PLCγ1-deficient GABAergic neurons, we crossed R26R-EYFP mice (Jax mouse cat No.006148) with *Plcg1*^*F/F*^*; Dlx5/6-Cre* mice and generated *Plcg1*^*F/F*^*; Dlx5/6-Cre;EYFP* mice. *Dlx5/6-Cre;EYFP* mice were used as controls in Fig. 3a–e. For the genetic deletion of PLCγ1 in excitatory neurons, *CaMKIIα-Cre* (MGI ID: 2176753) mice were bred to *Plcg1*^*F/F*^ mice to generate *Plcg1*^*F/F*^*; CaMKIIα-Cre* mice. Mice were maintained under standard housing conditions on a 12-hour light/dark cycle. Food and water were available *ad libitum*. All animal experiments were conducted according to protocols approved by Institutional Animal Care and Utilization Committee of Ulsan National Institute of Science and Technology. In our study, 10–16 months old mice (aged control and *Plcg1*^*F/F*^*; Dlx5/6-Cre* mice, male and female) were used throughout the behavioral tests (Fig. 1), *in vivo* EEG recording (Fig. 2), immunohistochemistry (Fig. 3), and *in vitro* brain slice electrophysiology experiment (Fig. 4). Young adult mice (8–12 weeks old, male and female) were also utilized for western blotting, fluorescence *in situ* hybridization, pilocarpine-induced seizure, additional locomotion and anxiety tests. The experimenter was not blind to the genotypes of animals.

### Tissue preparation and immunohistochemistry

Aged mice (10–16 months old) were deeply anaesthetized with avertin (250 mg/kg) and perfused transcardially with cold phosphate buffered saline followed by cold 4% paraformaldehyde. The dissected brains were fixed in 4% PFA at 4 °C overnight and then serially sectioned (40 μm) using a vibratome. Free-floating sections were permeabilized in 0.2% Triton X-100 in PBS for 1 hour and blocked in PBST containing 10% normal goat serum for 1 hour at room temperature. Sections were incubated in primary antibody solution diluted in PBST containing 10% NGS for overnight at 4 °C. Following three washes in PBS, sections were incubated with Alexa Fluor-tagged secondary antibodies at 1:400 dilution for 2 hours at room temperature. Primary antibodies used in this study were anti-Parvalbumin (Synaptic systems, 195 004, 1:1000), anti-Somatostatin (Santa cruz, sc-7819, 1:500), anti-GAD67 (Millipore, MAB5406, 1:200), anti-Gephyrin (Synaptic systems, 147 111, 1:200), anti-VGAT (Synaptic systems, 131 003, 1:200), anti-GFP (CiteAb, GFP-1010, 1:1000), and anti-NeuN (Sigma, MAB377, 1:1000). Secondary antibodies were Alexa Fluor 488 or Alexa Fluor 594 or Alexa Fluor 647-conjugated anti-rabbit, anti-mouse, anti-chicken, anti-guinea pig (Invitrogen, 1:400). All sections were counterstained with Hoechst 33258 (Sigma, 94403, 1:1000).

### Image acquisition and quantitative analysis

Images were derived from FV1000 confocal laser scanning microscope (Olympus) using UPLSAPO 10X NA:0.40, UPLSAPO 40 × 2 NA:0.95, UPLSAPO 60X O NA:1.35 objective lens and Axio zoom microscope (Carl Zeiss). Images were analyzed by ImageJ (NIH) program. Identical acquisition parameters were used for all the brain slices within a single set of experiment. Image analysis was not performed blind to the genotypes of animals. Inhibitory synapse was defined as co-localization between inhibitory presynaptic marker (VGAT) and inhibitory postsynaptic marker (Gephyrin). Puncta analyzer ver2.0 plugin for ImageJ was downloaded from github (https://github.com/physion/puncta-analyzer) and used to quantify the inhibitory synaptic puncta. The protocol using the Puncta analyzer is well described in the previous article^62^.

### Fluorescence *in situ* hybridization

Fluorescence *in situ* hybridization was conducted as previously described^63^. Frozen brain sections (14 μm thick) from young adult mice (8–12 weeks old) were cut coronally through the cortex and striatum formation. Sections were thaw-mounted onto Superfrost Plus Microscope Slides (Fisher Scientific #12-550-15). The sections were fixed in 4% paraformaldehyde for 10 min, dehydrated in increasing concentrations of ethanol for 5 min, and finally air-dried. Tissues were then pretreated for protease digestion for 10 min at room temperature. Probe hybridization and amplification were performed at 40 °C using HybEZ hybridization oven (Advanced Cell Diagnostics, Hayward, CA). The probes used in this study were three synthetic oligonucleotides complementary to the nucleotide (nt) sequence 1116–2000 of Mm-Plcg1-C2, nt 62–3113 of Mm-Gad1-C2, and nt 464–1415 of Mm-Slc17a7/Vglut1-C3 (Advanced Cell Diagnostics, Hayward, CA). The labeled probes were conjugated to Alexa Fluor 488, Atto 550, and Atto 647. The sections were hybridized with the labeled probe mixture at 40 °C for 2 hours per slide. Unbound hybridization probes were removed by washing the sections three times with 1x wash buffer at room temperature for 2 min. Following steps for signal amplification included incubations at 40 °C with Amplifier 1-FL for 30 min, with Amplifier 2-FL for 15 min, with Amplifier 3-FL for 30 min, and with Amplifier 4 Alt B-FL for 15 min. Each amplifier solution was removed by washing with 1x wash buffer at room temperature for 2 min. The slides were viewed, analyzed, and photographed using TCS SP8 Dichroic/CS (Leica).

### Western blot

Proteins were extracted from brain tissue of young adult mice (8 weeks old) using lysis buffer containing 1% Triton X-100, 10% glycerol, 150 mM NaCl, 50 mM HEPES, 2.5 mM sodium pyrophosphate (dibasic), 1 mM EGTA, 1 mM EDTA. Protease and phosphatase inhibitors including NaF, Aprotinin, Na_3_VO_4_, phenylmethylsulfonyl fluoride, Leupeptin, and pepstatin A were also freshly added. Bovine serum albumin was used to measure protein concentration by Bradford assay (BIO-RAD, 5000006). Proteins with sample buffer were denatured at 95 °C for 5 min. The same volume of samples was loaded in the gel, electrophoresed, and transferred into nitrocellulose membrane. Membranes were blocked by 5% skim milk in TTBS and incubated in primary antibodies at 4 °C for overnight. Primary antibodies used for western blotting were β-actin (GeneTex, 1:5000) and PLCγ1 (generated in lab^64^, 1:3000). Membranes were washed three times, each for 10 min and then incubated with secondary HRP-conjugated antibodies (VWR, 1:5000) for 1 hour at room temperature. After washing, ECL (GE Healthcare, RPN2235) was used to detect the protein band signal through ChemiDoc^TM^ (BIO-RAD). ImageJ was used for the quantification of protein bands.

### Electroencephalographic (EEG) recordings

Aged *Plcg1*^*F/F*^ and *Plcg1*^*F/F*^*; Dlx5/6-Cre* mice (10–14 months old) were used for EEG recordings. Mice were anesthetized with avertin (250 mg/kg) by intraperitoneal injection. Telemetry transmitter (TA10ETA-F20, ETA-F10) were implanted bilaterally through holes in the skull (AP −1.5 mm, ML ± 1.3 mm, directly on the dura mater overlying the hippocampus from each hemisphere) and covered with dental cement. After one week of recovery, EEG signal was monitored on freely moving mice using EEG recording system (Dataquest A.R.T, Data Sciences International). EEG data were acquired at a sampling rate of 500 Hz using DSI Dataquest A.R.T. system without a filter cutoff. Simultaneous long-term video could not be taken due to the limitation of equipment. Experimenter directly checked and monitored both behavioral seizure and EEG during daytime. To induce the seizure, mice were gently grabbed and handled once a day for a week. Electrographic EEG seizures were analyzed by using Neuroscore software (Data Sciences International).

### Brain slice electrophysiology

Aged *Plcg1*^*F/F*^ and *Plcg1*^*F/F*^*; Dlx5/6-Cre* mice (10–16 months old) were used for whole-cell patch clamp recording. After anesthetizing a mouse with halothane, the brain was isolated and the hippocampus was dissected. Hippocampal slices (300 μm thick) were prepared using a vibratome (Leica, VT1000S) in an ice-cold cutting solution containing (in mM) 234 sucrose, 2.5 KCl, 1.25 NaH_2_PO_4_, 24 NaHCO_3_, 11 glucose, 0.5 CaCl_2_, 10 MgSO_4_, saturated with 95% O_2_ and 5% CO_2_. The slices were recovered at 35 °C for one hour and later maintained at room temperature in a recovery artificial cerebrospinal fluid solution containing (in mM) 124 NaCl, 3 KCl, 1.25 NaH_2_PO_4_, 26 NaHCO_3_, 10 glucose, 6.5 MgSO_4_, 1 CaCl_2_, saturated with 95% O_2_ and 5% CO_2_. Following the recovery, the slice was transferred to a submerged recording chamber and continuously perfused with a recording aCSF solution containing (in mM) 124 NaCl, 3 KCl, 1.25 NaH_2_PO_4_, 26 NaHCO_3_, 10 glucose, 1.3 MgSO_4_, 2.5 CaCl_2_, saturated with 95% O_2_ and 5% CO_2_ at room temperature throughout the experiments. For miniature excitatory postsynaptic currents (mEPSCs), the recording aCSF was supplemented with 1 μM TTX and 50 μM picrotoxin. Whole-cell recordings of CA1 and CA3 neurons were made using pipettes of 3–7 MΩ resistance. The cells were held at −70 mV, and the internal solution contained (in mM) 125 CsMeSO_3_, 2.8 NaCl, 20 HEPES, 0.4 EGTA, 4 ATP-Mg, 0.5 GTP-Na_2_, 10 phosphocreatine-Na_2_, 5 QX314 (pH = 7.25 and osmolality = 285–290 mOsm). For miniature inhibitory postsynaptic currents (mIPSCs), the recording aCSF was supplemented with 1 μM TTX, 10 μM DNQX and 50 μM D-AP5. Whole-cell recordings of CA1 and CA3 neurons were made using pipettes of 3–7 MΩ resistance. The cells were held at −70 mV, and the internal solution contained (in mM) 134 CsCl, 2 MgCl_2_, 10 HEPES, 1 EGTA, 2 ATP-Mg, 0.5 GTP-Na_2_, 5 phosphocreatine-Na_2_ (pH = 7.25 and osmolality = 285–290 mOsm). Data were collected with a MultiClamp 700B amplifier (Molecular Devices), digitized at 10 kHz with a Digidata 1550 digitizer (Molecular Devices). pClamp10 software (Molecular Devices) was used for data acquisition and analysis. mEPSCs and mIPSCs were analyzed with MiniAnalysis software (Synaptosoft Inc) using a detection threshold of 8 pA.

For whole-cell patch clamp recording in young adult mice (8 weeks old), acute brain slices (300 μm) containing hippocampus were obtained from control and *Plcg1*^*F/F*^*; Dlx5/6-Cre* mice using tissue vibratome (VT1200S, Leica). After dissection, slices were recovered for 20 min at 34 °C and additional 40 min at room temperature in artificial cerebrospinal fluid (aCSF) containing 125 mM NaCl, 2.5 mM KCl, 1.25 mM NaH_2_PO_4_, 25 mM NaHCO_3_, 1 mM MgCl_2_, 2 mM CaCl_2_ and 15 mM glucose oxygenated with 95% O_2_ and 5% CO_2_ before recording. Whole-cell voltage clamp recordings were performed in the recording chamber perfused with aCSF containing 0.5 M TTX citrate and 25 μM D-AP5 at 30–31 °C. Recordings were made with glass pipettes (2.5–3.5 MΩ) filled with Cs^+^-based low Cl^−^ internal solution containing 135 mM CsMeSO_3_, 10 mM HEPES, 1 mM EGTA, 3.3 mM QX-314 chloride, 0.1 mM CaCl_2_, 4 mM Mg-ATP, 0.3 mM Na3-GTP, 8 mM Na_2_-phosphocreatine (pH 7.3 with CsOH). To obtain miniature postsynaptic currents in the same cell, membrane potential was first held at −70 mV (reversal potential of chloride, junction potential corrected) to measure miniature excitatory postsynaptic currents (mEPSCs) and then held at 0 mV (reversal potential of ionotropic glutamate receptors, junction potential corrected) to measure miniature inhibitory postsynaptic currents (mIPSCs). CA1 and CA3 neurons were identified by IR-DIC optics (Olympus BX51WI microscope). Voltage-clamp recording was performed using Multiclamp 700B (Molecular Devices) and signals were filtered at 2 kHz and digitized at 10 kHz. Recording data were monitored and acquired online by WinWCP (Strathclyde Electrophysiology Software). Clampfit 10.7 software (Molecular Devices) was used for data analysis offline. mEPSCs and mIPSCs were analyzed with Mini Analysis software (Synaptosoft Inc.) with the detection threshold 5 pA for mEPSCs and threshold 10 pA for mIPSCs.

### Seizure detection and assessment of seizure susceptibility by pilocarpine

To evoke and detect handling-induced behavioral seizures, mice (male and female) were gently handled by grasping the tail or scruff 2–3 times a week throughout the lifetime of mice. Behavioral seizure was scored by using Racine scale^17^. In pilocarpine-induced seizure experiment, young adult mice (8–12 weeks old) were first injected with N-methyl scopolamine bromide (1 mg/kg, Sigma S8502) before the injection of pilocarpine to inhibit the peripheral cholinergic effects. 15 minutes later, mice were treated with pilocarpine (300 mg/kg, Sigma P6503). After administration of pilocarpine, behavioral seizure score was measured using modified Racine scale for 2 hours: grade (1) stiffness and rigid posture; (2) head nodding; (3) partial forelimb clonus; (4) severe whole body continuous seizures; (5) falling, forelimb clonus and jumping (generalized motor convulsions). To cease the epileptic activity, diazepam (5 mg/kg, Dongwha pharm) was injected to mice. Mortality was comparable during pilocarpine-induced seizure between the genotypes in young adult mice.

### Behavior test

Behavior test was conducted at daytime (10:00 A.M–14:00 P.M) under dim light condition. Male mice were used in behavioral experiments and the ages of the mice are as follows: aged adult mice (10–16 months) for open field test, elevated plus maze, Y maze, contextual fear conditioning, tail suspension test, and forced swim test (Fig. 1); young adult mice (8–12 weeks) for open field test and elevated plus maze (Fig. S2). To minimize the effects of stress on behavioral outcome, we started with the least stressful test and continued to perform other tests in the following order: elevated plus maze, open field test, Y-maze, contextual fear conditioning, tail suspension test, forced swim test. After behavioral test, mice were used in other experiments including immunohistochemistry, EEG recording, and brain slice electrophysiology.

#### Open-field test

Open field test was performed in a square white chamber (length 45 cm × width 45 cm × height 40 cm) under indirectly low illumination. Mice were gently placed in center region (20 cm × 20 cm) and allowed to freely explore for 60 min. The movements were recorded and analyzed by SMART video tracking system (Panlab).

#### Elevated plus maze

The elevated plus maze consisted of four arms, elevated 50 cm above the floor. Two arms were enclosed by 15 cm height walls, whereas two arms were open without walls. Mice were gently placed in center region and allowed to freely explore for 10 min. Overall activity was recorded and analyzed by SMART video tracking system.

#### Y maze

The white acryl maze consisted of three arms (length 35 cm × width 4 cm × height 15 cm). Three arms were extended from center region at equally 120° angle. In indirect, low lighting, mice were placed into center region and allowed to freely move through maze for 10 min. The spontaneous alternation was calculated with the following equation: The spontaneous alternation (%) = [(number of spontaneous alternations)/(total number of arm entries - 2)] × 100.

#### Contextual fear conditioning

Contextual fear conditioning was conducted in fear conditioning chamber (Panlab, Barcelona, Spain). In training session, mice were allowed to freely explore conditioning chamber for 2 min. After 2 min, sound pulse (amplitude 1, frequency 400 Hz) was given for 30 seconds and 0.8 mA electric footshock was delivered for 2 sec. After 24 hours, mice were put in the same conditioning chamber to test contextual fear memory for 3 min. The freezing time was recorded and analyzed by software (Panlab, Freezing 2.0 version).

#### Tail suspension test

Mice were suspended by adhesive taping their tail to suspension apparatus (length 50 cm × width 15 cm × height 30 cm, Harvard). The immobility time was recorded for 6 min, analyzed by SMART video tracking system, and manually counted by experimenter.

#### Forced swim test

The apparatus was a glass cylinder filled with water (25 °C, 20 cm diameter, 30 cm height). Mice were placed into cylinder and swimming behavior was recorded by SMART video tracking system for 6 min. The immobility time in last 4 min was scored manually.

### Statistics

We performed all statistical analyses using GraphPad Prism software (version 7, GraphPad Software). The appropriate statistical methods for each experiment were described in the figure legend and Supplementary Table. Summary graphs were all shown as mean ± SEM. Chi-square test, unpaired student t-test, two-way ANOVA with post-hoc Sidak’s multiple comparison test, two-way repeated measures ANOVA with post-hoc Sidak’s multiple comparison test were used to determine statistical differences between the genotypes. P value (<0.05) was considered as statistically significant. *P < 0.05, **P < 0.01, ***P < 0.001, ****P < 0.0001. The details of statistical analysis were presented in the Supplementary Table.

## Supplementary information

Supplementary information

Behavioral seizure

## Data Availability

The datasets produced during the current study are available from the corresponding author on request.
