# Supplementary material for: Deletion of PLCγ1 in GABAergic neurons increases seizure susceptibility in aged mice
Source: Sci Rep. 2019 Nov 28;9:17761. doi: 10.1038/s41598-019-54477-4 (PMC6882884; doi:10.1038/s41598-019-54477-4)
Supplement: Supplementary file 1 — Supplementary information [file 41598_2019_54477_MOESM1_ESM.pdf]

Supplementary Information for

Deletion of PLCγ1 in GABAergic neurons increases seizure susceptibility in aged mice

Hye Yun Kim[1], Yong Ryoul Yang[2], Hongik Hwang[3], Ha-Eun Lee[1], Hyun-Jun Jang[1], Jeongyeon Kim[4], Esther Yang[5], Hyun Kim[5], Hyewhon Rhim[3], Pann-Ghill Suh[1,4,*] and Jae-Ick Kim[1,*]

Affiliations

[1]School of Life Sciences, Ulsan National Institute of Science and Technology (UNIST), Ulsan 44919, Republic of Korea

[2]Aging Research Center, Korea Research Institute of Bioscience and Biotechnology (KRIBB), Daejeon 34141, Republic of Korea

[3]Center for Neuroscience, Brain Science Institute, Korea Institute of Science and Technology (KIST), Seoul 136-791, Republic of Korea

[4]Korea Brain Research Institute (KBRI), Daegu 41062, Republic of Korea

[5]Department of Anatomy, College of Medicine, Korea University, Seoul, 136-705, Republic of Korea

*Corresponding authors.

Supplementary Figure 1

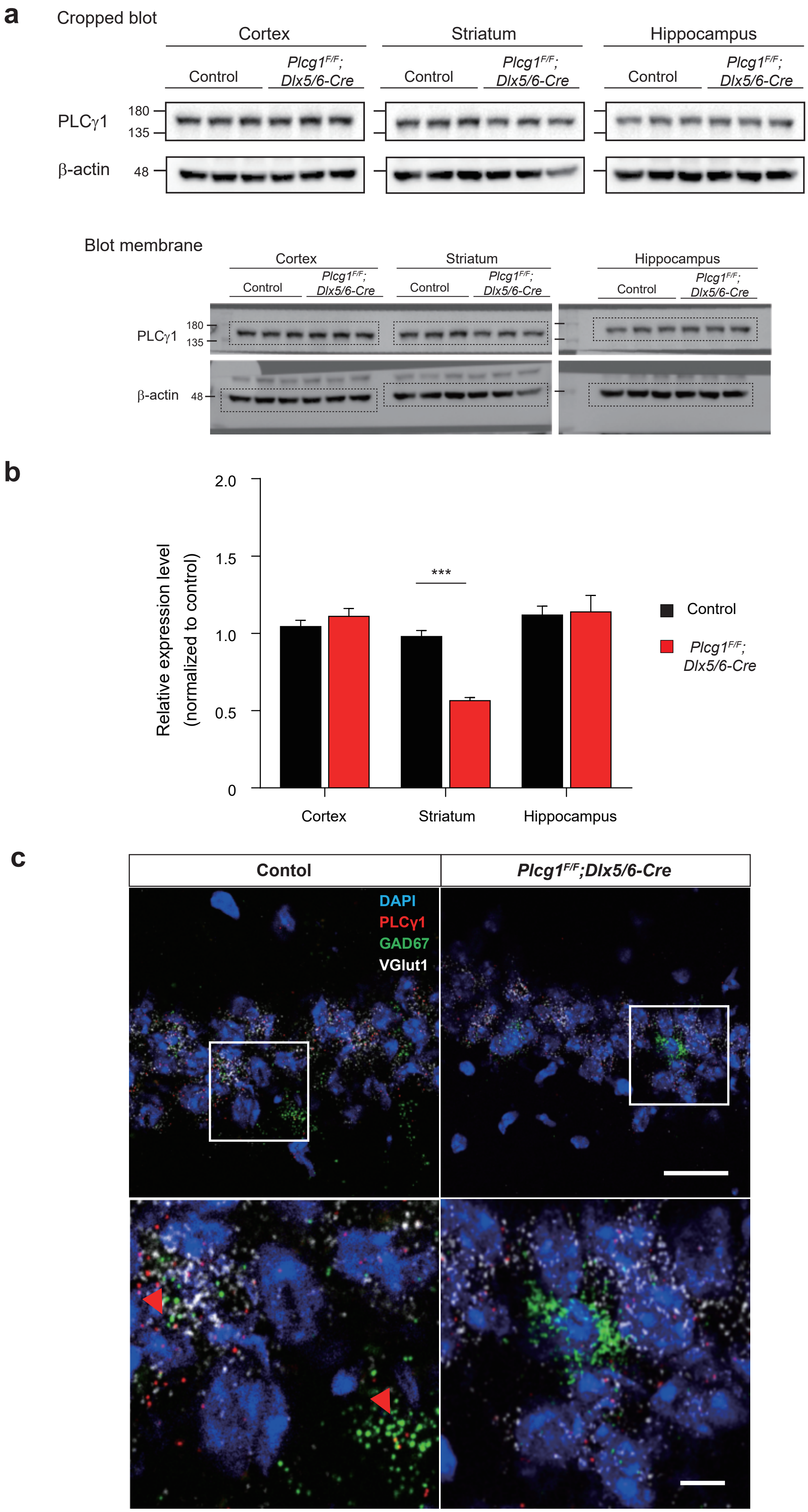

Supplementary Figure 1. PLCγ1 is selectively deleted in GABAergic neurons of *Plcg1$^{F/F}$;Dlx5/6-Cre* mice.

(a) Expression of PLCγ1 in several brain sub-regions of control and *Plcg1$^{F/F}$;Dlx5/6-Cre* mice confirmed by western blot assay (top). Uncropped blot image of PLCγ1 and β-actin (bottom). β-actin blot was derived from same gel of PLCγ1 western blot. Exposure time: 16 s (PLCγ1) and 60 s (β-actin). (b) Quantification of PLCγ1 expression normalized to control (n = 3 for each genotype, unpaired t-test, ***P = 0.0007 for striatum, P > 0.05 for other brain regions). (c) Cell-type specific PLCγ1 knockout at RNA level in CA1 region of hippocampus by fluorescence *in situ* hybridization. Scale bar: 25 μm (top), 5 μm (bottom). All data were expressed as mean ± SEM.

Supplementary Figure 2

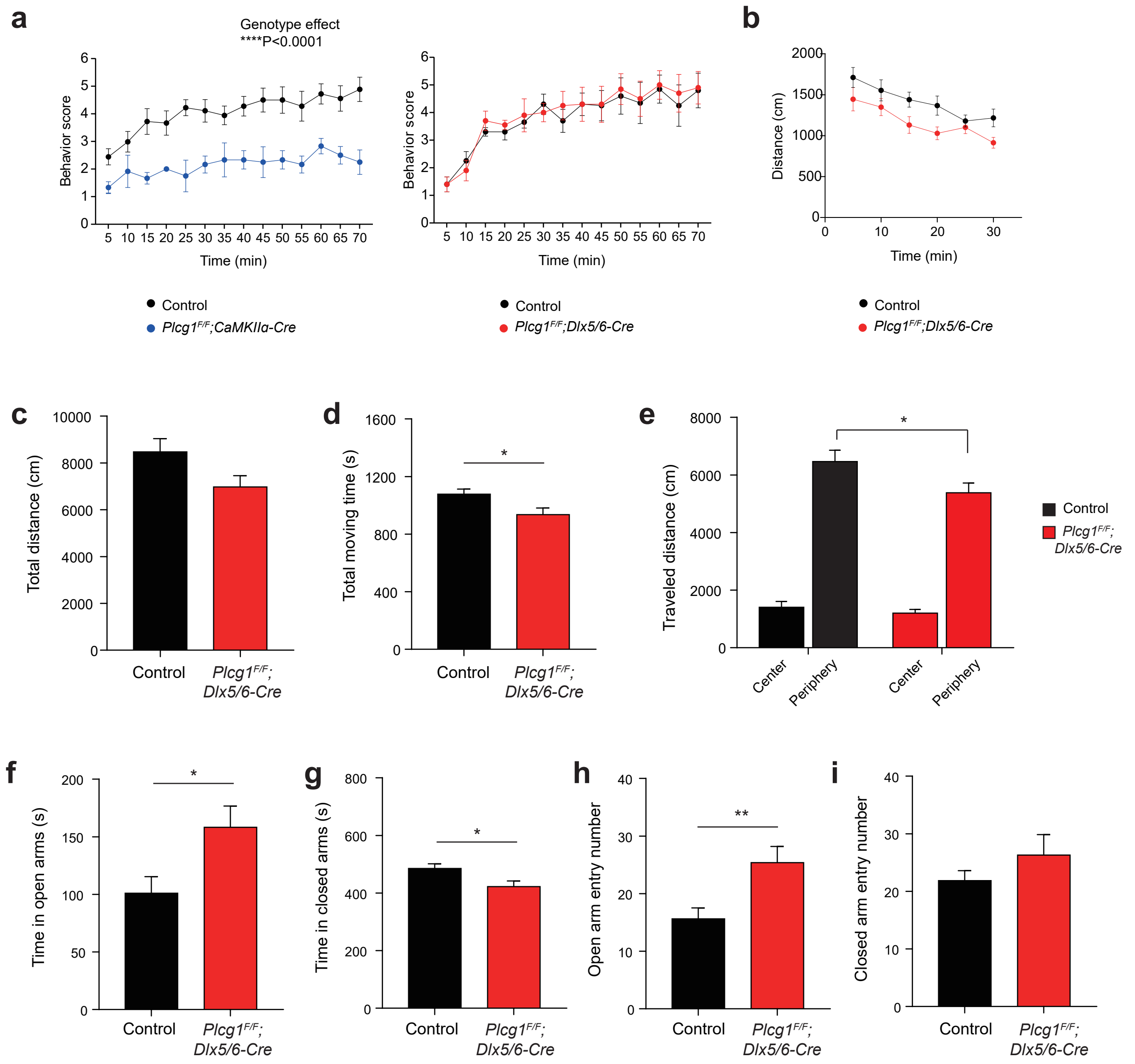

Supplementary Figure 2. Young adult *Plcg1$^{F/F}$;Dlx5/6-Cre* mice begin to show behavioral alterations in locomotion and anxiety.

(a) Behavior seizure score in pilocarpine-induced seizure model at 10 - 12 weeks old (control n = 9, *Plcg1$^{F/F}$;CaMKIIα-Cre*, n = 6, two-way repeated measures ANOVA, genotype effect, ****P < 0.0001; control n = 10, *Plcg1$^{F/F}$;Dlx5/6-Cre*, n = 10; two-way repeated measures ANOVA, genotype effect, P > 0.05). (b) Distance travelled for 30 minutes in open filed test (control, n = 13; *Plcg1$^{F/F}$;Dlx5/6-Cre*, n = 11; two-way repeated measures ANOVA, genotype effect, P = 0.0614). (c, d) Total distance travelled (c) and moving time (d) in open-field test (unpaired t-test, $P_{distance}$ = 0.0614, *P_{time} = 0.0190). (e) Thigmotaxis in open-field test (two-way repeated measures ANOVA, genotype effect, P = 0.0886, interaction, *P = 0.0423, *$P_{periphery}$ = 0.0245 in Sidak's multiple comparison test). (f, g) Time spent in open (f) and closed arms (g) of elevated plus maze (control, n = 13; *Plcg1$^{F/F}$;Dlx5/6-Cre*, n = 11; unpaired t-test, *P_{open} = 0.0215, *P_{closed} = 0.0196). (h, i) Entry number in open (h) and closed arms (i) of elevated plus maze (unpaired t-test, **P_{open} = 0.0072, P_{closed} = 0.2383). All data were expressed as mean ± SEM.

Supplementary Figure 3

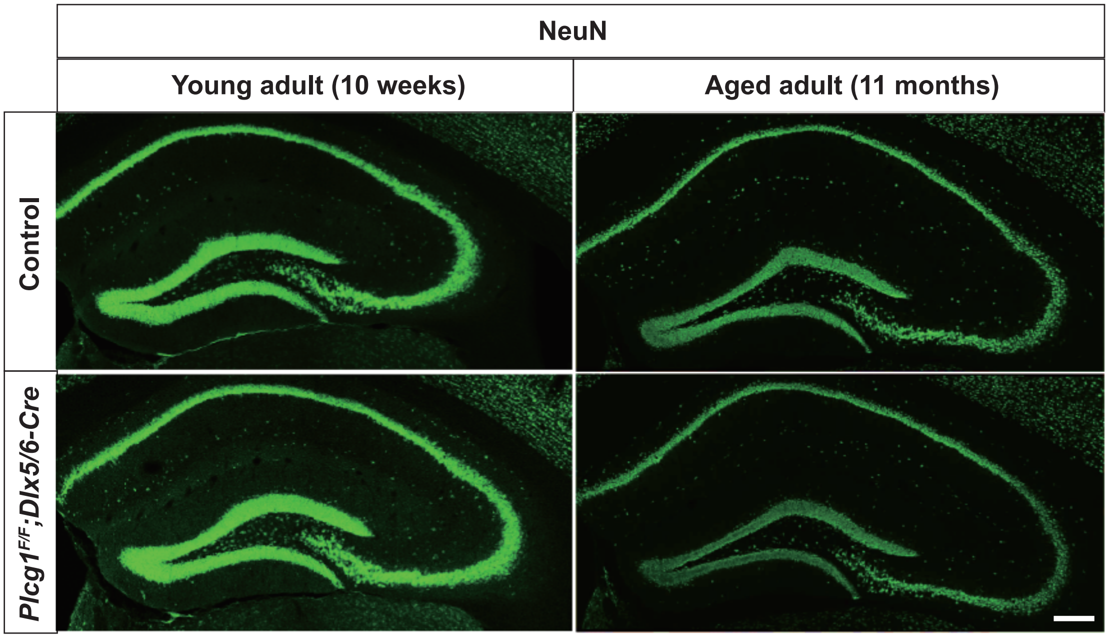

Supplementary Figure 3. There is no overall structural change in the hippocampus between the genotypes.

The overall hippocampal structure in young and aged mice between control and *Plcg1*$^{F/F}$*;Dlx5/6-Cre* mice with neuronal marker NeuN. Scale bar: 200 μm.

Supplementary Figure 4

a CA3 region of the hippocampus in control mice

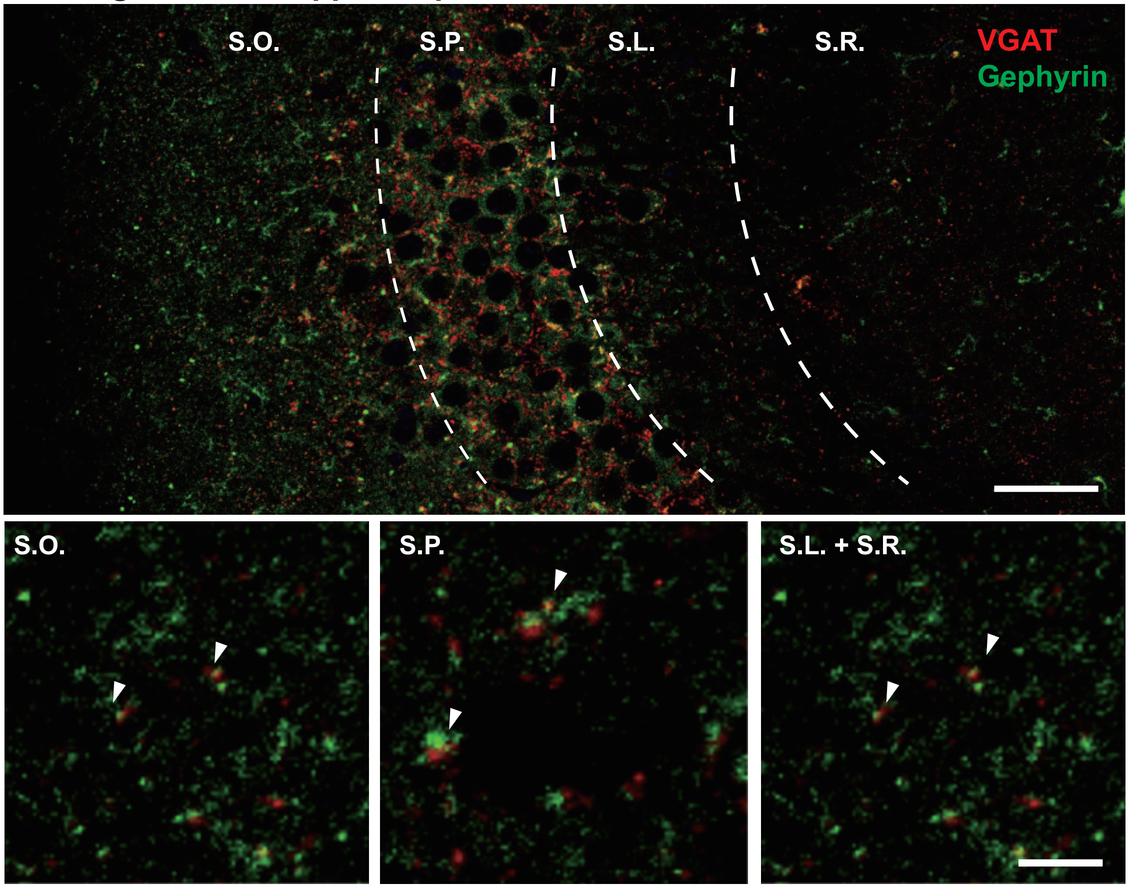

b

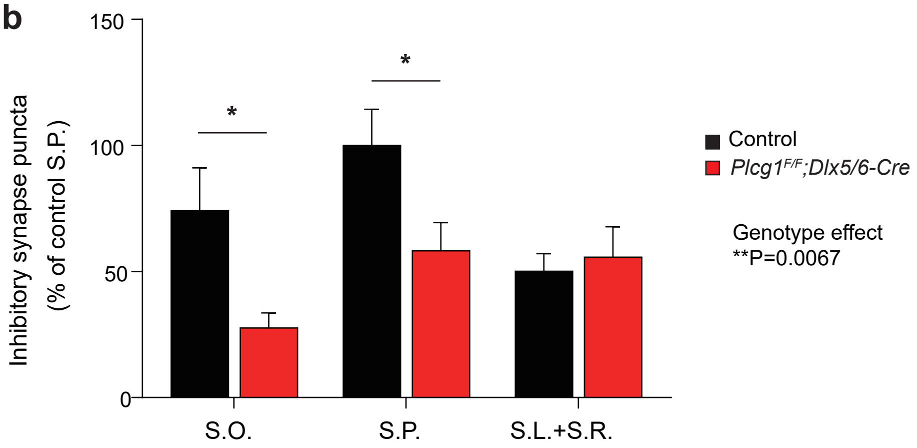

Supplementary Figure 4. Both somatic inhibitory synapses and dendritic inhibitory synapses are decreased in aged *Plcg1$^{F/F}$;Dlx5/6-Cre* mice.

(a) Representative image of inhibitory synapses in specific layers of hippocampal CA3 region in control mouse. White arrowhead: co-localization of VGAT and gephyrin. Scale bar: 50 μm (top), 5 μm (bottom). (b) Quantification of inhibitory synapse number in specific layers of CA3 (control n = 28 from 6 mice, *Plcg1$^{F/F}$;Dlx5/6-Cre*, n = 25 slices from 6 mice, two-way ANOVA, genotype effect, **P = 0.067, *$P_{S.O.}$ = 0.0206, *$P_{S.P.}$ = 0.0450 by post-hoc test).

Supplementary Figure 5

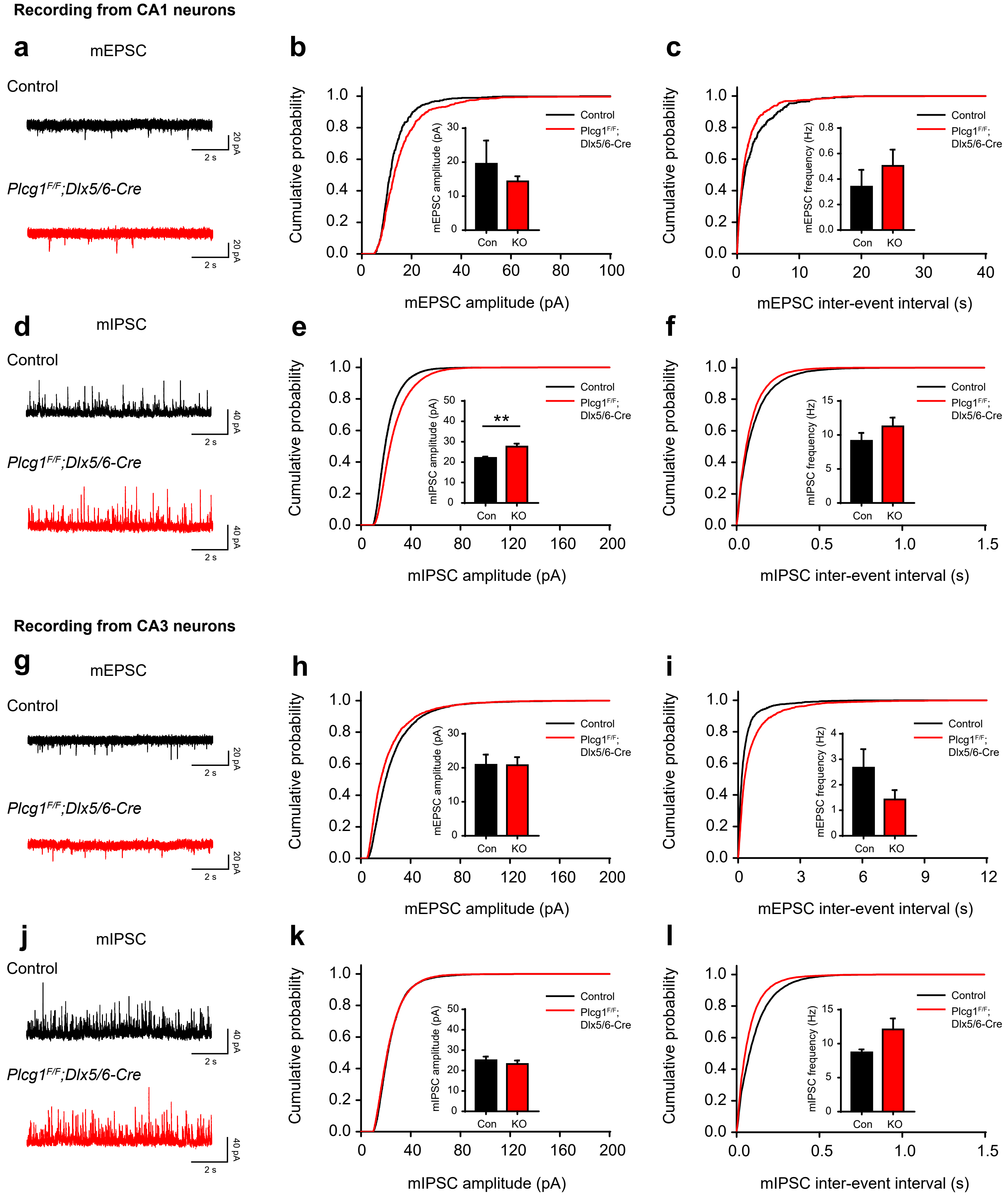

Supplementary Figure 5. Young *Plcg1$^{F/F}$;Dlx5/6-Cre* mice show increased amplitude of mIPSC in CA1 region of the hippocampus.

(a) Representative recording trace of mEPSCs recorded from hippocampal CA1 region in young age (8 weeks old) control and *Plcg1$^{F/F}$;Dlx5/6-Cre* mice (control, n = 7, *Plcg1$^{F/F}$;Dlx5/6-Cre*, n = 9). (b, c) Quantification of mEPSCs amplitude (b) and frequency (c) in CA1. (d) Representative recording trace of mIPSCs recorded from hippocampal CA1 region in young age control and *Plcg1$^{F/F}$;Dlx5/6-Cre* mice (control, n = 8, *Plcg1$^{F/F}$;Dlx5/6-Cre*, n = 7). (e, f) Quantification of mIPSCs amplitude (e) and frequency (f) in CA1 (amplitude, unpaired t-test, **P = 0.0031). (g) Representative recording trace of mEPSCs recorded from hippocampal CA3 region in young age control and *Plcg1$^{F/F}$;Dlx5/6-Cre* mice (control, n = 8, *Plcg1$^{F/F}$;Dlx5/6-Cre*, n = 10). (h, i) Quantification of mEPSCs amplitude (h) and frequency (i) in CA3. (j) Representative recording trace of mIPSCs recorded from hippocampal CA3 region in young age control and *Plcg1$^{F/F}$;Dlx5/6-Cre* mice (control, n = 7, *Plcg1$^{F/F}$;Dlx5/6-Cre*, n = 9). (k, l) Quantification of mIPSCs amplitude (k) and frequency (l) in CA3. All data were expressed as mean ± SEM.

Supplementary Table S1

FIGURE		DESCRIPTION	VALUE<br>(Average ± SEM)	N number	STATISTIC	P VALUE	POST TEST	POST TEST<br>P VALUE
1	c	Incidence of seizures	15.38%<br>75.00%	2 of 13 mice<br>12 of 16 mice	Chi-square	0.0014	N/A	N/A
	g	Control<br>*Plcg1*$^{F/F}$;*Dlx5/6-Cre*	N/A	N = 9 mice<br>N = 11 mice	Repeated measures 2-way ANOVA<br>Interaction, F (5, 85) = 0.06178<br>Time effect, F (5, 85) = 53.48<br>Genotype effect, F (1, 17) = 8.27	Interaction<br>0.9974<br>Time<br>< 0.0001<br>Genotype<br>0.0105	Sidak's multiple comparisons test	10min: 0.1504<br>20min: 0.0712<br>30min: 0.1075<br>40min: 0.2223<br>50min: 0.1363<br>60min: 0.1652
	h	Control<br>*Plcg1*$^{F/F}$;*Dlx5/6-Cre*	20863 ± 1068 cm<br>17389 ± 583 cm	N = 9 mice<br>N = 11 mice	Unpaired t-test<br>t(18) = 3	0.0077	N/A	N/A
	i	Control<br>*Plcg1*$^{F/F}$;*Dlx5/6-Cre*	2364 ± 94.61 s<br>1990 ± 41.94 s	N = 9 mice<br>N = 11 mice	Unpaired t-test<br>t(18) = 3	0.0012	N/A	N/A
	j	Control Center<br>Control Periphery<br>*Plcg1*$^{F/F}$;*Dlx5/6-Cre* Center<br>*Plcg1*$^{F/F}$;*Dlx5/6-Cre* Periphery	3541 ± 477.70 cm<br>17322 ± 719.80 cm<br>2530 ± 203.70 cm<br>14859 ± 619.10 cm	N = 9 mice (Con)<br>N = 11 mice (cKO)	Repeated measures 2-way ANOVA<br>Interaction, F (1, 18) = 2.311<br>Genotype effect, F (1, 18) = 9<br>Region effect F, (1, 18) = 746	Genotype<br>0.0077	Sidak's multiple comparisons test	Center<br>0.3387<br>Periphery<br>0.0046
	l	Control<br>*Plcg1*$^{F/F}$;*Dlx5/6-Cre*	104.50 ± 12.31 s<br>185.10 ± 11.83 s	N =12 mice (Con)<br>N = 9 mice (cKO)	Unpaired t-test<br>t(19) = 4.594	0.0002	N/A	N/A
	m	Control<br>*Plcg1*$^{F/F}$;*Dlx5/6-Cre*	165.90 ± 13.23 s<br>86 ± 12.37 s		Unpaired t-test<br>t(19) = 4.273	0.0004	N/A	N/A
	n	Control<br>*Plcg1*$^{F/F}$;*Dlx5/6-Cre*	15.25 ± 1.68<br>22.67 ± 1.24		Unpaired t-test<br>t(19) = 3.345	0.0034	N/A	N/A
	o	Control<br>*Plcg1*$^{F/F}$;*Dlx5/6-Cre*	15.25 ± 1.15<br>10.67 ± 1.28		Unpaired t-test<br>t(19) = 2.65	0.0158	N/A	N/A
	q	Control<br>*Plcg1*$^{F/F}$;*Dlx5/6-Cre*	50.10 ± 6.76 %<br>30.69 ± 5.77 %	N = 8 mice<br>N = 11 mice	Unpaired t-test<br>t(17) = 2.183	0.0433	N/A	N/A
	r	Control<br>*Plcg1*$^{F/F}$;*Dlx5/6-Cre*	35.73 ± 2.35 %<br>33.31 ± 2.44 %	N = 8 mice<br>N = 11 mice	Unpaired t-test<br>t(17) = 0.6932	0.4975	N/A	N/A
	s	Control<br>*Plcg1*$^{F/F}$;*Dlx5/6-Cre*	27.74 ± 8.65 %<br>26.12 ± 5.76 %	N = 8 mice<br>N = 11 mice	Unpaired t-test<br>t(17) = 0.8725	0.8725	N/A	N/A
	t	Control<br>*Plcg1*$^{F/F}$;*Dlx5/6-Cre*	41.88 ± 8.23 %<br>38 ± 6.12 %	N = 8 mice<br>N = 11 mice	Unpaired t-test<br>t(17) = 0.3865	0.7039	N/A	N/A
2	d	**Gamma**<br>Control<br>*Plcg1*$^{F/F}$;*Dlx5/6-Cre*<br>**Delta**<br>Control<br>*Plcg1*$^{F/F}$;*Dlx5/6-Cre*<br>**Theta**<br>Control<br>*Plcg1*$^{F/F}$;*Dlx5/6-Cre*<br>**Alpha**<br>Control<br>*Plcg1*$^{F/F}$;*Dlx5/6-Cre*<br>**Beta**<br>Control<br>*Plcg1*$^{F/F}$;*Dlx5/6-Cre*	**Gamma**<br>0.002376 ± 0.0009669 V^2<br>0.01386 ± 0.005488 V^2<br>**Delta**<br>0.0004495 ± 0.0001511 V^2<br>0.00195 ± 0.0006828 V^2<br>**Theta**<br>0.0004173 ± 0.0001557 V^2<br>0.001365 ± 0.0005037 V^2<br>**Alpha**<br>0.0003175 ± 5.068e-005 V^2<br>0.001109 ± 0.0003875 V^2<br>**Beta**<br>0.0008645 ± 0.0002663 V^2<br>0.004332 ± 0.00163 V^2	N = 4 mice (Con)<br>N = 6 mice (cKO)	Repeated measures 2-way ANOVA<br>Interaction, F (4, 40) = 1.915<br>Frequency effect, F (4, 40) = 3.649<br>Genotype effect, F (1, 40) = 6.22	Genotype<br>0.0169	Sidak's multiple comparisons test	Gamma 0.0054

3	b	**DG**<br>Control<br>$Plcg1^{F/F}$*;Dlx5/6-Cre*<br>**CA3**<br>Control<br>$Plcg1^{F/F}$*;Dlx5/6-Cre*<br>**CA1**<br>Control<br>$Plcg1^{F/F}$*;Dlx5/6-Cre*	**DG**<br>77.03 ± 3.12<br>69.19 ± 3.29<br>**CA3**<br>33.41 ± 1.59<br>28 ± 1.10<br>**CA1**<br>54 ± 2.61<br>46.17 ± 2.74	N = 34 slices from 6 mice (Con)<br>N = 36 slices from 6 mice (cKO)	Repeated measures 2-way ANOVA<br>Interaction, F (2, 136) = 0.2899<br>Region effect, F (2, 136) = 267.2<br>Genotype effect, F (1, 68) = 5.862	Genotype<br>0.0181	Sidak's multiple comparisons test	DG<br>0.0913<br>CA3<br>0.4012<br>CA1<br>0.0914
	d	**DG**<br>Control<br>$Plcg1^{F/F}$*;Dlx5/6-Cre*<br>**CA3**<br>Control<br>$Plcg1^{F/F}$*;Dlx5/6-Cre*<br>**CA1**<br>Control<br>$Plcg1^{F/F}$*;Dlx5/6-Cre*	**DG**<br>13.39 ± 1.14<br>16.72 ± 0.61<br>**CA3**<br>14.11 ± 1.44<br>11.61 ± 0.53<br>**CA1**<br>18.83 ± 0.88<br>19.06 ± 1.34	N = 18 slices from 3 mice per each genotype	Repeated measures 2-way ANOVA<br>Interaction, F (2, 68) = 4.574<br>Region effect, F (2, 68) = 20.38<br>Genotype effect, F (1, 34) = 0.1301	Interaction<br>0.0137<br>Region<br>< 0.0001<br>Genotype<br>0.7205	Sidak's multiple comparisons test	DG<br>0.0775<br>CA3<br>0.2574<br>CA1<br>0.9983
	e	**DG**<br>Control<br>$Plcg1^{F/F}$*;Dlx5/6-Cre*<br>**CA3**<br>Control<br>$Plcg1^{F/F}$*;Dlx5/6-Cre*<br>**CA1**<br>Control<br>$Plcg1^{F/F}$*;Dlx5/6-Cre*	**DG**<br>39.39 ± 2.03<br>36 ± 1.82<br>**CA3**<br>21.56 ± 1.13<br>17.83 ± 0.97<br>**CA1**<br>34.17 ± 1.44<br>28.94 ±2.04	N = 18 slices from 3 mice per each genotype	Repeated measures 2-way ANOVA<br>Interaction, F (2, 68) = 0.2015<br>Region effect, F (2, 68) = 70.75<br>Genotype effect, F (1, 34) = 7.926	Interaction<br>0.8180<br>Region<br>< 0.0001<br>Genotype<br>0.0081	Sidak's multiple comparisons test	DG<br>0.3720<br>CA3<br>0.2917<br>CA1<br>0.0739
	g	**DG**<br>Control<br>$Plcg1^{F/F}$*;Dlx5/6-Cre*<br>**CA3**<br>Control<br>$Plcg1^{F/F}$*;Dlx5/6-Cre*<br>**CA1**<br>Control<br>$Plcg1^{F/F}$*;Dlx5/6-Cre*	**DG**<br>20.33 ± 1.67<br>16.31 ± 1.19<br>**CA3**<br>19.4 ± 2.29<br>17.06 ± 0.51<br>**CA1**<br>15.22 ± 1.64<br>11.41 ± 0.81	N = 5 slices from 4 mice (Con)<br>N = 6 slices from 3 mice (cKO)	2-way ANOVA<br>Interaction, F (2, 26) = 0.2231<br>Region effect, F (2, 26) = 9.392<br>Genotype effect, F (1, 26) = 9.661	Interaction<br>0.8016<br>Region<br>0.0009<br>Genotype<br>0.0045	Sidak's multiple comparisons test	DG<br>0.1127<br>CA3<br>0.5689<br>CA1<br>0.1407
	i	**DG**<br>Control<br>$Plcg1^{F/F}$*;Dlx5/6-Cre*<br>**CA3**<br>Control<br>$Plcg1^{F/F}$*;Dlx5/6-Cre*<br>**CA1**<br>Control<br>$Plcg1^{F/F}$*;Dlx5/6-Cre*	**DG**<br>100 ± 4.47 %<br>76.65 ± 4.21 %<br>**CA3**<br>112.30 ± 6.66 %<br>87.82 ± 4.13 %<br>**CA1**<br>112.20 ± 4.72 %<br>95.48 ± 3.60 %	N = 28 slices from 6 mice (Con)<br>N = 20 slices from 6 mice (cKO)	2-way ANOVA<br>Interaction, F (2, 140) = 0.3427<br>Region effect, F (2, 140) = 5.133<br>Genotype effect, F (1, 140) = 26.92	Interaction<br>0.7104<br>Region<br>0.0071<br>Genotype<br>< 0.0001	Sidak's multiple comparisons test	DG<br>0.0037<br>CA3<br>0.0027<br>CA1<br>0.0662
	j	**DG**<br>Control<br>$Plcg1^{F/F}$*;Dlx5/6-Cre*<br>**CA3**<br>Control<br>$Plcg1^{F/F}$*;Dlx5/6-Cre*<br>**CA1**<br>Control<br>$Plcg1^{F/F}$*;Dlx5/6-Cre*	**DG**<br>100 ± 3.41 %<br>94.48 ±2.30 %<br>**CA3**<br>129.40 ± 4.93 %<br>124.90 ± 3.74 %<br>**CA1**<br>113.90 ± 4.29 %<br>106.50 ± 3.52 %	N = 28 slices from 6 mice (Con)<br>N = 20 slices from 6 mice (cKO)	2-way ANOVA<br>Interaction, F (2, 140) = 0.06646<br>Region effect, F (2, 140) = 27.82<br>Genotype effect, F (1, 140) = 3.087	Interaction<br>0.9357<br>Region<br>< 0.0001<br>Genotype<br>0.0811	Sidak's multiple comparisons test	DG<br>0.6965<br>CA3<br>0.8235<br>CA1<br>0.4897

	k	**DG**<br>Control<br>*Plcg1$^{F/F}$;Dlx5/6-Cre*<br>**CA3**<br>Control<br>*Plcg1$^{F/F}$;Dlx5/6-Cre*<br>**CA1**<br>Control<br>*Plcg1$^{F/F}$;Dlx5/6-Cre*	**DG**<br>100 ± 13.77 %<br>38.79 ± 10.31 %<br>**CA3**<br>123.40 ± 20.65 %<br>67.34 ± 16.29 %<br>**CA1**<br>114.20 ± 18.12 %<br>67.18 ± 17.47 %	N = 28 slices from 6 mice (Con)<br>N = 24 slices from 6 mice (cKO)	2-way ANOVA<br>Interaction, F (2, 154) = 0.09356<br>Region effect, F (2, 154) = 1.42<br>Genotype effect, F (1, 154) = 16.39	Interaction<br>0.9107<br>Region<br>0.2448<br>Genotype<br>< 0.0001	Sidak's multiple comparisons test	DG<br>0.0260<br>CA3<br>0.0538<br>CA1<br>0.1409
4	b	Control<br>*Plcg1$^{F/F}$;Dlx5/6-Cre*	17.44 ± 0.83 pA<br>16.29 ± 0.80 pA	N = 12 cells<br>N = 9 cells	Unpaired t-test<br>t(19) = 0.9756	0.3415	N/A	N/A
	c	Control<br>*Plcg1$^{F/F}$;Dlx5/6-Cre*	0.46 ± 0.05 Hz<br>0.32 ± 0.04 Hz	N = 12 cells<br>N = 9 cells	Unpaired t-test<br>t(19) = 1.953	0.0657	N/A	N/A
	e	Control<br>*Plcg1$^{F/F}$;Dlx5/6-Cre*	28.83 ± 1.60 pA<br>29.53 ± 2.24 pA	N = 10 cells<br>N = 12 cells	Unpaired t-test<br>t(12) = 1.41	0.8074	N/A	N/A
	f	Control<br>*Plcg1$^{F/F}$;Dlx5/6-Cre*	6.84 ± 0.38 Hz<br>6.90 ± 0.60 Hz	N = 10 cells<br>N = 12 cells	Unpaired t-test<br>t(12) = 1.085	0.9329	N/A	N/A
	h	Control<br>*Plcg1$^{F/F}$;Dlx5/6-Cre*	16.62 ± 1.35 pA<br>14.08 ± 1.01 pA	N = 8 cells<br>N = 6 cells	Unpaired t-test<br>t(20) = 0.247	0.1839	N/A	N/A
	i	Control<br>*Plcg1$^{F/F}$;Dlx5/6-Cre*	0.57 ± 0.12 Hz<br>0.36 ± 0.14 Hz	N = 8 cells<br>N = 6 cells	Unpaired t-test<br>t(20) = 0.08531	0.2990	N/A	N/A
	k	Control<br>*Plcg1$^{F/F}$;Dlx5/6-Cre*	30.47 ± 3.28 pA<br>27.73 ± 2.06 pA	N = 6 cells<br>N = 9 cells	Unpaired t-test<br>t(13) = 0.7492	0.4671	N/A	N/A
	l	Control<br>*Plcg1$^{F/F}$;Dlx5/6-Cre*	6.08 ± 0.81 Hz<br>3.75 ± 0.63 Hz	N = 6 cells<br>N = 9 cells	Unpaired t-test<br>t(13) = 2.311	0.0379	N/A	N/A
S1	b	**Cortex**<br>Control<br>*Plcg1$^{F/F}$;Dlx5/6-Cre*<br>**Striatum**<br>Control<br>*Plcg1$^{F/F}$;Dlx5/6-Cre*<br>**Hippocampus**<br>Control<br>*Plcg1$^{F/F}$;Dlx5/6-Cre*	**Cortex**<br>1.04 ± 0.04<br>1.11 ± 0.05<br>**Striatum**<br>0.98 ± 0.04<br>0.57 ± 0.02<br>**Hippocampus**<br>1.12 ± 0.06<br>1.14 ± 0.11	3 mice per each genotype	Unpaired t-test<br>Cortex<br>t(4) = 1.041<br>Striatum<br>t(4) = 9.475<br>Hippocampus<br>t(4) = 0.1702	Cortex<br>0.3565<br>Striatum<br>0.0007<br>Hippocampus<br>0.8731	N/A	N/A
S2	a	Pilocarpine-induced seizure assessment	N/A	Set 1<br>Control, N = 9 mice<br>*Plcg1$^{F/F}$;CaMKIIα-Cre,* N = 6 mice	Repeated measures 2-way ANOVA<br>Interaction, F (13, 169) = 0.5127<br>Time effect, F (13, 169) = 3.372<br>Genotype effect, F (1, 13) = 41.17	Interaction<br>0.9147<br>Time<br>0.0001<br>Genotype<br>< 0.0001	N/A	N/A
				Set 2<br>Control, N = 10 mice<br>*Plcg1$^{F/F}$;Dlx5/6-Cre,* N = 10 mice	Repeated measures 2-way ANOVA<br>Interaction, F (13, 234) = 0.2609<br>Time effect, F (13, 234) = 15.27<br>Genotype effect, F (1, 18) = 0.03515	Interaction<br>0.9958<br>Time<br>< 0.0001<br>Genotype<br>0.8534	N/A	N/A
	c	Control<br>*Plcg1$^{F/F}$;Dlx5/6-Cre*	8472 ± 564.40 cm<br>6973 ± 487.60 cm	N =13 mice<br>N =11 mice	Unpaired t-test<br>t(22) = 1.971	0.0614	N/A	N/A
	d	Control<br>*Plcg1$^{F/F}$;Dlx5/6-Cre*	1078 ± 34.73 s<br>936 ± 45.34 s	N =13 mice<br>N =11 mice	Unpaired t-test<br>t(22) = 2.531	0.0190	N/A	N/A

	e	Control Center<br>Control Periphery<br>*Plcg1*$^{F/F}$*;Dlx5/6-Cre* Center<br>*Plcg1*$^{F/F}$*;Dlx5/6-Cre* Periphery	1406 ± 205 cm<br>6464 ± 392 cm<br>1201 ± 130 cm<br>5384 ± 336 cm	N =13 mice<br>N =11 mice	Repeated measures 2-way ANOVA<br>Interaction, F (1, 22) = 4.646<br>Genotype effect, F (1, 22) = 3.175<br>Region effect, F (1, 22) = 518.8	Interaction<br>0.0423<br>Genotype effect<br>0.0886<br>Region effect<br>< 0.0001	Sidak's multiple comparisons test	Center<br>0.8575<br>Periphery<br>0.0245
	f	Control<br>*Plcg1*$^{F/F}$*;Dlx5/6-Cre*	101 ± 14.48 s<br>158.30 ± 18.39 s	N = 13 mice<br>N =10 mice	Unpaired t-test<br>t(21) = 2.485	0.0215	N/A	N/A
	g	Control<br>*Plcg1*$^{F/F}$*;Dlx5/6-Cre*	485.60 ± 15.89 s<br>422.10 ± 19.84 s	N = 13 mice<br>N =10 mice	Unpaired t-test<br>t(21) = 2.527	0.0196	N/A	N/A
	h	Control<br>*Plcg1*$^{F/F}$*;Dlx5/6-Cre*	15.62 ± 1.92<br>25.40 ± 2.81	N = 13 mice<br>N =10 mice	Unpaired t-test<br>t(21) = 2.976	0.0072	N/A	N/A
	i	Control<br>*Plcg1*$^{F/F}$*;Dlx5/6-Cre*	21.85 ± 1.73<br>26.30 ± 3.54	N = 13 mice<br>N =10 mice	Unpaired t-test<br>t(21) = 1.214	0.2383	N/A	N/A
S4	b	**S.O.**<br>Control<br>*Plcg1*$^{F/F}$*;Dlx5/6-Cre*<br>**S.P.**<br>Control<br>*Plcg1*$^{F/F}$*;Dlx5/6-Cre*<br>**S.L.+S.R.**<br>Control<br>*Plcg1*$^{F/F}$*;Dlx5/6-Cre*	**S.O.**<br>74.12 ±16.96 %<br>27.57 ±6.02 %<br>**S,P.**<br>100 ± 14.35 %<br>58.28 ± 11.11 %<br>**S.L.+S.R.**<br>50.07 ± 7.03 %<br>55.75 ± 12.03 %	N = 28 slices from 6 mice (Con)<br>N = 24 slices from 6 mice (cKO)	2-way ANOVA<br>Interaction, F (2, 148) = 2.677<br>Region, F (2, 148) = 3.398<br>Genotype, F (1, 148) = 7.577	Interaction<br>0.0721<br>Region<br>0.0361<br>Genotype<br>0.0067	Sidak's multiple comparisons test	S.O.<br>0.0206<br>S.P.<br>0.0450<br>S.L.+S.R.<br>0.9848
S5	b	Control<br>*Plcg1*$^{F/F}$*;Dlx5/6-Cre*	19.54 ± 6.82 pA<br>14.37 ± 1.49 pA	N = 7 cells<br>N = 9 cells	Unpaired t-test<br>t(14) = 0.8331	0.4188	N/A	N/A
	c	Control<br>*Plcg1*$^{F/F}$*;Dlx5/6-Cre*	0.34 ± 0.13 Hz<br>0.50 ± 0.13 Hz	N = 7 cells<br>N = 9 cells	Unpaired t-test<br>t(14) = 0.88	0.3937	N/A	N/A
	e	Control<br>*Plcg1*$^{F/F}$*;Dlx5/6-Cre*	22.07 ± 0.68 pA<br>27.64 ± 1.46 pA	N = 8 cells<br>N = 7 cells	Unpaired t-test<br>t(13) = 3.626	0.0031	N/A	N/A
	f	Control<br>*Plcg1*$^{F/F}$*;Dlx5/6-Cre*	9.14 ± 1.17 Hz<br>11.27 ± 1.29 Hz	N = 8 cells<br>N = 7 cells	Unpaired t-test<br>t(13) = 1.226	0.2420	N/A	N/A
	h	Control<br>*Plcg1*$^{F/F}$*;Dlx5/6-Cre*	20.89 ± 3.00 pA<br>20.77 ± 2.36 pA	N = 8 cells<br>N = 10 cells	Unpaired t-test<br>t(16) = 0.03033	0.9762	N/A	N/A
	i	Control<br>*Plcg1*$^{F/F}$*;Dlx5/6-Cre*	2.67 ± 0.73 Hz<br>1.43 ± 0.37 Hz	N = 8 cells<br>N = 10 cells	Unpaired t-test<br>t(16) = 1.628	0.1231	N/A	N/A
	k	Control<br>*Plcg1*$^{F/F}$*;Dlx5/6-Cre*	25.05 ± 1.82 pA<br>23.24 ± 1.70 pA	N = 7 cells<br>N = 9 cells	Unpaired t-test<br>t(14) = 0.7229	0.4816	N/A	N/A
	l	Control<br>*Plcg1*$^{F/F}$*;Dlx5/6-Cre*	8.72 ± 0.44 Hz<br>12.09 ± 1.62 Hz	N = 7 cells<br>N = 9 cells	Unpaired t-test<br>T(14) = 1.782	0.0965	N/A	N/A
